# Vitamin D Metabolism and Profiling in Veterinary Species

**DOI:** 10.3390/metabo10090371

**Published:** 2020-09-15

**Authors:** Emma A. Hurst, Natalie Z. Homer, Richard J. Mellanby

**Affiliations:** 1 The Roslin Institute and Royal (Dick) School of Veterinary Studies, The University of Edinburgh, Easter Bush, Midlothian, Edinburgh, Scotland EH25 9RG, UK; Richard.Mellanby@ed.ac.uk; 2 Mass Spectrometry Core, Edinburgh Clinical Research Facility, Queen’s Medical Research Institute, The University of Edinburgh, Little France Crescent, Edinburgh, Scotland EH16 4TJ, UK; n.z.m.homer@ed.ac.uk

**Keywords:** vitamin D, 25-hydroxyvitamin-D, 1,25-dihydroxyvitamin-D, 24,25-dihydroxyvitamin-D, C3-epimers, free vitamin D, LC-MS/MS, veterinary, profiling, comparative

## Abstract

The demand for vitamin D analysis in veterinary species is increasing with the growing knowledge of the extra-skeletal role vitamin D plays in health and disease. The circulating 25-hydroxyvitamin-D (25(OH)D) metabolite is used to assess vitamin D status, and the benefits of analysing other metabolites in the complex vitamin D pathway are being discovered in humans. Profiling of the vitamin D pathway by liquid chromatography tandem mass spectrometry (LC-MS/MS) facilitates simultaneous analysis of multiple metabolites in a single sample and over wide dynamic ranges, and this method is now considered the gold-standard for quantifying vitamin D metabolites. However, very few studies report using LC-MS/MS for the analysis of vitamin D metabolites in veterinary species. Given the complexity of the vitamin D pathway and the similarities in the roles of vitamin D in health and disease between humans and companion animals, there is a clear need to establish a comprehensive, reliable method for veterinary analysis that is comparable to that used in human clinical practice. In this review, we highlight the differences in vitamin D metabolism between veterinary species and the benefits of measuring vitamin D metabolites beyond 25(OH)D. Finally, we discuss the analytical challenges in profiling vitamin D in veterinary species with a focus on LC-MS/MS methods.

## 1. Introduction

Vitamin D is well known to play an important role in skeletal health and disease. Vitamin D deficiency, however, is prevalent worldwide [1,2] and is associated with a myriad of health disorders, including many outwith its well-recognized role in the musculoskeletal system [2]. Nonetheless, there remains controversy surrounding optimal levels of vitamin D for maintaining the health of human and veterinary patients, and recommendations for daily vitamin D requirements [3,4,5,6,7,8]. Currently vitamin D status is assessed by measuring the concentration of 25-hydroxyvitamin-D (25(OH)D), owing to its relative abundance in the circulation, ease of analysis, stability, and half-life. Extensive reviews on profiling vitamin D metabolites beyond 25(OH)D in humans have recently been published [9,10]. However, there is still limited knowledge of this extensive pathway in veterinary species [11,12,13,14], and importantly, defined reference ranges for even the routinely measured 25(OH)D are lacking in most veterinary species. Some differences in veterinary vitamin D metabolism are recognized; for example, some carnivorous species including dogs and cats cannot produce vitamin D cutaneously [15,16,17,18]. Numerous studies have shown that vitamin D deficiency is associated with skeletal and non-skeletal disease in companion animals [19,20,21,22,23,24,25,26,27,28,29] and reproductive outcomes in farm animals [30].

An extensive network of vitamin D metabolites that contribute to the functional activity and catabolism of vitamin D in a range of diseases and tissue types are now being identified [3,9,31,32]. With improving technology, namely liquid chromatography tandem mass spectrometry (LC-MS/MS), that can facilitate the accurate identification and quantification of multiple highly similar metabolites in a single sample, it is now possible to profile the vitamin D pathway more extensively than ever before in veterinary patients [32,33,34,35,36]. Doing so in health and disease will enable us to further our understanding of this pathway, identify new biomarkers which can improve clinical diagnostics and may enable a treat-to-target approach of vitamin D supplementation. In this review, we will discuss vitamin D metabolism and profiling in veterinary species, highlighting differences in vitamin D metabolism between species and the benefits of profiling beyond 25(OH)D in veterinary clinical diagnostics and therapeutics. Finally, we will discuss the analytical challenges of profiling vitamin D metabolites in veterinary species with a focus on LC-MS/MS technology.

## 2. Fundamentals of Vitamin D Metabolism

**Figure 1:**
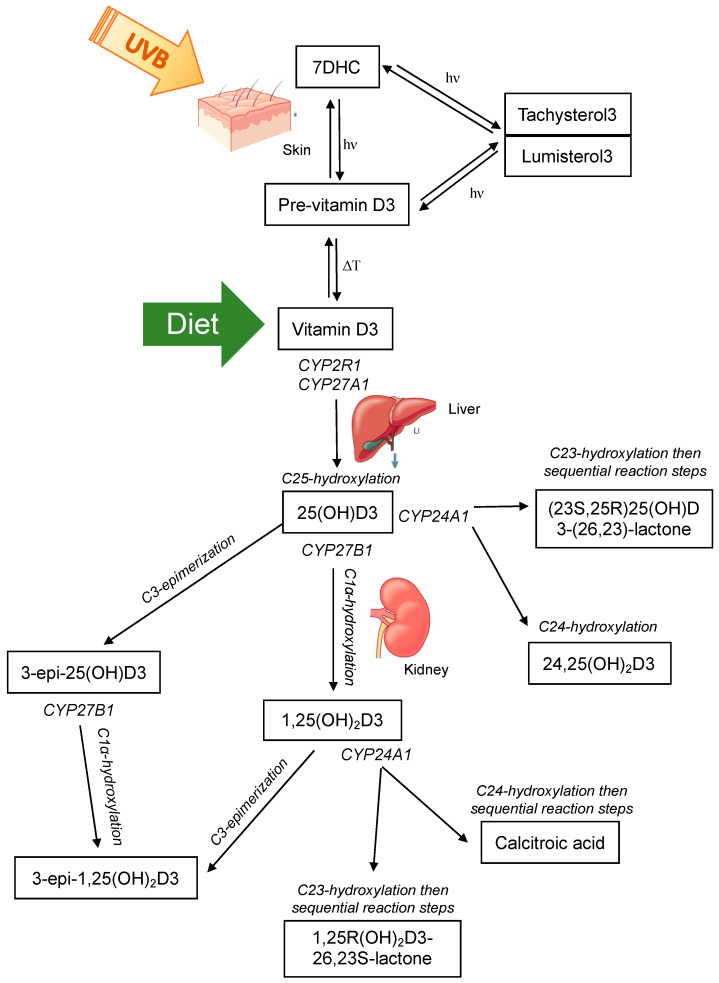
The major classical vitamin D pathway, whereby vitamin D3 is either produced in the skin via photochemical (hν) conversion of 7-dehydrocholesterol (7DHC) to pre-vitamin D3 and subsequent thermoisomerization (ΔT) to vitamin D3, or consumed in the diet. Vitamin D3 is hydroxylated at C25 in the liver by CYP enzymes and then subsequently subject to further hydroxylation or C3-epimerization in the kidney. Note that vitamin D2 is activated by CYP2R1 and CYP27B1 and can undergo C24 hydroxylation and C3-epimerization as shown for vitamin D3, but is only consumed through the diet and not endogenously produced.

Vitamin D is present in two main forms, D2 and D3. Vitamin D2 is synthesized by the action of ultraviolet B (UVB) radiation on ergosterol in fungi and yeast (often found in small amounts on plants) [37,38,39] and is consumed from a plant-based diet. Vitamin D3 is synthesized in the skin of humans and some animals by the action of UVB radiation (280–320 nm) on 7-dehydrocholesterol (7DHC) [40,41,42], or can be consumed from animal products. Upon exposure of the skin to UVB radiation, a photochemical reaction occurs, resulting in the production of pre-vitamin D3 from 7DHC. Subsequently, a reversible thermoisomerization reaction occurs, slowly converting pre-vitamin D3 into vitamin D3 [40,43,44]. Alternatively, pre-vitamin D3 can be further photoisomerized into inert isomers (lumisterol and tachysterol), or reversed back into 7DHC. Each reaction requires a different UV action spectrum range [41,45] (Figure 1).

Both vitamin D2 and D3 enter the circulation and are predominately bound to the vitamin D binding protein (VDBP) with a small percentage also bound to albumin, and less than 1% circulating as free or unbound [46,47,48]; other vitamin D metabolites are also bound in this manner. Vitamin D2/3 are prohormones that are subsequently activated by sequential hydroxylation at C25 in the liver to 25-hydroxyvitamin-D2/3 (25(OH)D2/3), and then at C1α to the most hormonally active form 1α,25-dihydroxyvitamin-D2/3 (1,25(OH)_2_D2/3) (Figure 1). These hydroxylation steps occur by the actions of the cytochrome P450 (CYP) enzyme family [49]. Hydroxylation at C25 is primarily catalyzed by 25-hydroxylases, CYP2R1 in the endoplasmic reticulum of the liver and to a lesser extent by CYP27A1 in the mitochondria of hepatocytes [50,51]. Hydroxylation at C1α occurs in the mitochondria of proximal convoluted tubule cells of the kidney by the action of 1α-hydroxylase CYP27B1 [52]. This enzyme has been detected in other tissues and cell types and evidence of local production of 1,25(OH)_2_D2/3 was a major contributor to identifying extraskeletal roles of vitamin D [53,54,55]. 1,25(OH)_2_D exerts its actions on target cells and tissues by binding to the nuclear vitamin D receptor (VDR) and heterodimerizing with retinoid X receptor (RXR). This complex exerts genomic actions as a transcription factor to regulate target genes that contain a vitamin D response element in their promoter. Alternatively, 1,25(OH)_2_D can bind to the plasma membrane VDR and induce non-genomic actions, for example, the stimulation of intestinal calcium transport [40,56].

The primary role of 1,25(OH)_2_D is the maintenance of calcium and phosphate homeostasis. Therefore, the regulation of CYP27B1 activity is tightly controlled via parathyroid hormone (PTH) [57,58,59] and fibroblast growth factor 23 (FGF23) [60], as well as a negative feedback loop whereby 1,25(OH)_2_D acts on itself to suppress CYP27B1 and induce its own catabolism by promoting CYP24A1 activity [58,61]. CYP24A1 can induce C23- and C24-hydroxylation of 1,25(OH)_2_D and 25(OH)D [49,62,63,64] (Figure 1). Whether the predominant hydroxylation step occurs at the 23- or 24-carbon position is determined by the residue at position 326 of CYP24A1 [64]. When alanine at position 326 (favours C24-hydroxylation) is substituted for a glycine (favours C23-hydroxylation), the side chain of the substrate can dock further into the binding pocket of the enzyme, placing C23 rather than C24 into the optimal position for hydroxylation [64]. This change gives rise to the C24-hydroxylation pathway being altered to favour the C23-hydroxylation pathway. C24-hydroxylation of 1,25(OH)_2_D results in a five-step process that culminates with calcitroic acid; and the C23-hydroxylation pathway involves conversion of 1,25(OH)_2_D3 to 1,25R(OH)_2_D3-26,23S-lactone through sequential hydroxylation steps [65]. The end products are physiologically different; calcitroic acid is rapidly excreted in the bile with no discernible biological activity, whereas 1,25R(OH)_2_D3-26,23S-lactone belongs to a family of known VDR antagonists with superior VDBP binding and greater metabolic stability [64,66]. C23-hydroxylation of 25(OH)D3 forms 23,25(OH)_2_D3, with subsequent steps forming the end product (23S,25R)25(OH)D3-(26,23)-lactone. C24-hydroxylation of 25(OH)D3 forms 24,25-dihyroxyvitamin-D3 (24,25(OH)_2_D23). 24,25(OH)_2_D3 was thought to be an inactive catabolic product of 25(OH)D3, however studies have now shown that it exerts biological activity independent of the VDR [67,68,69,70]. Interestingly, the occurrence of C23 versus C24 hydroxylation by CYP24A1 is species dependent [64,66,71,72,73,74]. Some species, such as humans, use both pathways; others preferentially 23-hydroxylate (the opossum and the guinea pig) or 24-hydroxylate (the rat) [66]. The functional significance of the two distinct pathways in different species is unknown. However, the metabolic products of the 1,25R(OH)_2_D3-26,23S-lactone pathway may provide a protective mechanism to attenuate any vitamin D challenge as they are strong VDR antagonists, suggesting that species with CYP24A1 containing Gly-326 that favours the C23-hydroxylation pathway may better adapt to excessive 1,25(OH)_2_D3 activation or excessive dietary calcium or phosphorus [64,66,75,76].

## 3. Vitamin D Metabolism in Veterinary Species

**Figure 2:**
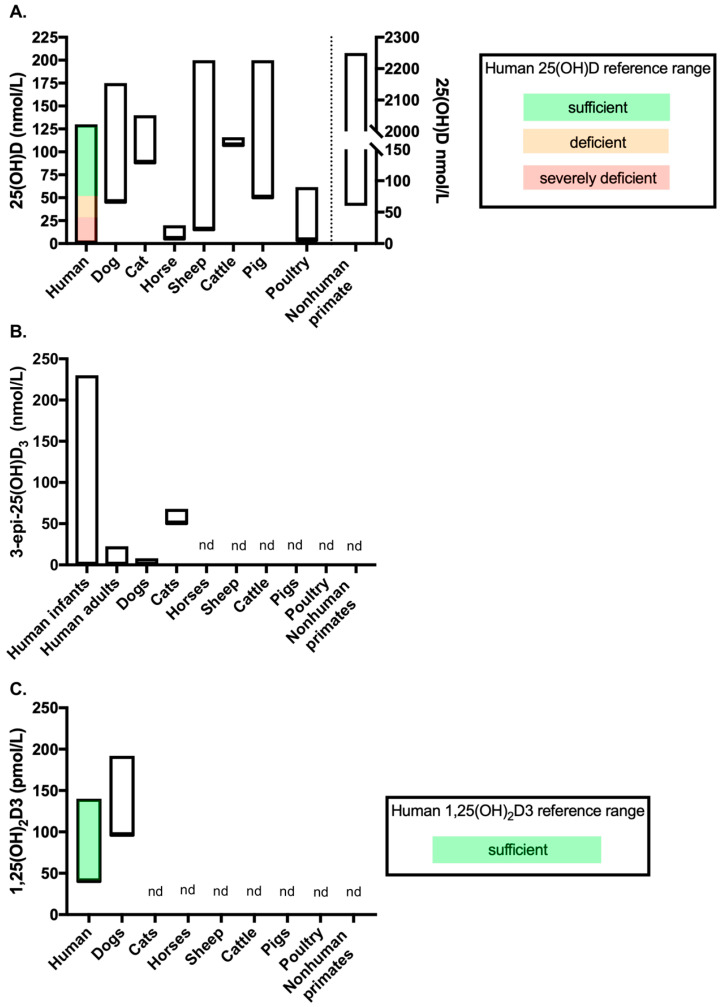
Vitamin D metabolite concentrations across species measured by LC-MS/MS (data from the studies included in Table 1). Figure showing the range of (**A**) 25(OH)D, (**B**) 3-epi-25(OH)D3 and (**C**) 1,25(OH)_2_D3 concentrations measured in healthy adult controls of each species by LC-MS/MS. Where available, reference ranges are highlighted by coloured bars. The human 25(OH)D and 1,25(OH)_2_D3 reference ranges are from the NHS website (accessed September 2020). No reference range exists for 3-epi-25(OH)D3 in humans, therefore data from a systemic review which reported ranges from several studies was used (see Bailey et al. (2013) [79]). The number of studies included to provide data for each of the veterinary species are as follows: (**A**) dogs n = 8 [80,81,82,83,84,85,86,87], cats n = 1 [85], horses n = 1 [88], sheep n = 4 [30,89,90,91], cattle n = 2 [92,93], pigs n = 1 [94], poultry n = 2 [95,96] and nonhuman primates n = 2 [97,98]; (**B**) dogs n = 2 [81,85] and cats n = 1 [85]; and **C**. dogs n = 1 [83]. nd = no data available for the metabolites measured by LC-MS/MS in that species. In chart (**A**), data for nonhuman primates’ measures against the right-hand Y-axis.

The associations between vitamin D status and disease in animal species has been extensively reviewed elsewhere [11,12,13,14], as have the comparative aspects of metabolic bone diseases related to vitamin D in animal species [77]. This review section will therefore focus on highlighting differences in vitamin D metabolism between animal species and the potential benefits of assessing vitamin D metabolites beyond 25(OH)D in animal health and disease. For reference, Figure 2 displays vitamin D metabolite concentration ranges across healthy adult animals of the species discussed below, compared to human reference ranges or human data for those metabolites as a comparison. In order to enable a direct comparison, this figure only includes data from studies outlined in Table 1 (see Section 4 introduction) in which vitamin D metabolites were measured by LC-MS/MS (studies discussed below include other methods of measuring vitamin D metabolites, however they were not included in Figure 2).

### 3.1. Comparative Differences in Vitamin D Metabolism

Evolutionary, genetic and environmental factors have influenced vitamin D metabolism across animal species. Through adaptations to specific diets and environments, the source and specific functions of vitamin D as mediators of calcium and phosphorus homeostasis differs across species. Although deficiencies in vitamin D can result in diseases with very similar pathology, there are clear differences in vitamin D metabolism and thus the specific factors inducing disease must not be assumed to be the same across species [77,78]; therefore, this must be a key factor when considering preventative and therapeutic methods. Here we will discuss key differences in vitamin D metabolism between species.

#### 3.1.1. Dogs and Cats

Likely through evolutionary adaptation to a carnivorous, vitamin D rich diet, dogs and cats have a reduced ability to synthesize vitamin D cutaneously via dermal photosynthesis in comparison to herbivore species. The consumption of prey animals, particularly of fat, liver, and blood, which is high in vitamin D, and of meat, which is very high in phosphorus, provided adequate nutritional supply of vitamin D. The reduced ability to synthesize vitamin D in the skin of these animals is due to high activity of the enzyme 7-dehydrocholesterol-reductase, which converts 7DHC into cholesterol, reducing concentrations of the precursor for photochemical conversion into pre-vitamin D3 [16,17,18]. As such, pet dogs and cats now rely upon dietary vitamin D. As expected by the lack of cutaneous vitamin D production, vitamin D status in dogs does not show seasonal variation in line with changes in UVB exposure in temperate regions, as it does in species that do produce vitamin D cutaneously [80]. An initial study by Griffiths et al. in 1988 showed that husky dogs in the Antarctic actually demonstrated an inverse relationship between UVB radiation and 25(OH)D concentration [99]; later, Laing et al. (1999) studied greyhounds in Australia and demonstrated a lack of seasonal fluctuation [100]. More recently, a longitudinal study that followed 18 dogs over a one-year period and were fed a standardized diet, demonstrated that vitamin D status, as defined by measurement of both 25(OH)D2 and 25(OH)D3 concentration by LC-MS/MS, does not exhibit seasonal fluctuation [80]. To the authors knowledge, no such longitudinal studies have been completed in cats.

Such reliance on dietary vitamin D makes it important that cats and dogs consume adequate amounts of vitamin D in their diet. Feeding pets’ homemade diets that are not certified by a veterinary professional, for example, may not be nutritionally adequate [101]. Although not common, there have been instances of vitamin D toxicity in these species as a result of over supplementation of commercial foodstuff [102,103]. Interestingly, carnivore species are considered more resistant to vitamin D toxicity in comparison to omnivores due to their ingestion of large amounts [101], however numerous cases of vitamin D toxicity in dogs and cats have resulted in many animals requiring veterinary intervention [102,103,104,105]. The concentration of vitamin D required to induce toxicity in dogs is unknown. In one study, a commercial dog food supplemented with over 100 times the recommended amount resulted in severe vitamin D toxicity [104]; whereas another study demonstrated that supplementation with five times the recommended dose did not significantly increase serum 25(OH)D concentrations of the study population [106]. Other occasions of vitamin D over supplementation have not specified vitamin D concentrations [102,105,107].

There are noteworthy differences in vitamin D metabolism that apply specifically to cats. It has been demonstrated that cats cannot use vitamin D2 (the plant derived version) as efficiently as vitamin D3, with vitamin D2 supplementation less able to elevate plasma vitamin D and 25(OH)D concentrations compared to vitamin D3 supplementation [108]. This is thought to be due, in part, to a lower binding affinity of VDBP to D2 metabolites in comparison to D3, perhaps as a consequence of a strict carnivorous diet resulting in historical dietary intake of vitamin D2 being low [11]. Conversely, dogs have been shown to use both D2 and D3 efficiently [12]. In dogs fed commercial dog food, levels of serum 25(OH)D2 are rarely detectable due to predominant supplementation with vitamin D3.

Another unique difference in cats is that the C3-epimerization pathway is quantitatively significant [85] (Figure 2B). The C3-epimer was detected in cats at much higher concentrations than reported in other species, with the mean concentration reported as 58.7 nmol/L (23.5 ng/mL) in a study by Sprinkle et al. (2018) which used high performance liquid chromatography (HPLC) to quantify the vitamin D analytes. In the same study, rats had a mean 3-epi-25(OH)D3 concentration of 3.3 nmol/L (1.3 ng/mL) and dogs had undetectable 3-epi-25(OH)D3, where the method had a limit of detection (LOD) of 12.5 nmol/L (5 ng/mL) [85]. Interestingly, when the cats were switched to a different diet that contained four times more vitamin D3 per kg of body weight compared to the initial diet, serum 25(OH)D3 concentrations were not significantly different but the concentration of 3-epi-25(OH)D3 increased significantly, as did the percentage contribution of the epimer to total 25(OH)D3 [85]. The author discusses the C3-epimerization pathway as a potential protective mechanism in cats, which may explain their theorized increased resistance to vitamin D toxicity. A previous study has shown that cats supplemented with dietary vitamin D3 at 63 times the recommended amount did not show clinical signs of vitamin D toxicosis despite serum levels of 25(OH)D3 reaching 1071.8 nmol/L (429.4 ng/mL) [109]. Recently, a study investigating 3-epi-25(OH)D3 concentrations in dogs using LC-MS/MS, demonstrated that 3-epi-25(OH)D3 could be detected in 87.2% of the 117 dogs tested, with a mean concentration of 5.2 nmol/L (2.1 ng/mL) and a lower limit of quantitation (LLOQ) of 4 nmol/L (1.6 ng/mL) [81]. The concentration of 3-epi-25(OH)D3 in dogs detected in this study is comparable to the low levels detected in other species and much lower than those detected in cats by Sprinkle et al. (Figure 2B). This finding supports the argument that dogs may be more susceptible to vitamin D toxicosis than cats due to the relative lack of activation of the C3-epimerization pathway. Although the study by Sprinkle et al. measured only a small number of cats, the potential high levels of 3-epi-25(OH)D3 in cats should be taken into consideration when analyzing cat samples for 25(OH)D3. Many methods do differentiate between 25(OH)D3 and 3-epi-25(OH)D3, therefore high levels of 3-epi-25(OH)D may result in overestimation of 25(OH)D3 if they are not separated and individually quantified.

#### 3.1.2. Horses

Horses very rarely develop rickets [13,110] and this is likely due to them having evolved a unique mechanism for calcium homeostasis that is quite different to other animals. Horses have lower concentrations of circulating vitamin D (both 25(OH)D and 1,25(OH)_2_D) than other veterinary species [111,112,113,114]; in fact, the concentrations are so low that in other species they would be considered vitamin D deficient (Figure 2A). In comparison to other species, horses exhibit high intestinal calcium absorption, high renal calcium excretion and high blood calcium levels, with low vitamin D metabolite concentrations and a decreased sensitivity of the parathyroid gland to calcium [111,112,115,116,117]. Calcium homeostasis is less dependent on vitamin D in horses than in other species; low VDR expression in the small intestine and kidney coupled with high intestinal absorption of calcium and low vitamin D concentrations supports this conclusion [118]. Wilkens et al. (2017) have described novel regulatory proteins for the regulation of transepithelial calcium transport in the small intestine of the horse that are independent of vitamin D [114]. Azarpeykan et al. (2016) have, however, demonstrated a statistically significant correlation between calcium transporting genes and expression of the VDR in the equine kidney, suggesting that even low concentrations of 1,25(OH)_2_D may tightly regulate vitamin D responsive calcium transport in horses [119]. Azarpeykan et al. (2016) also highlight differences in CYP enzyme expression in the horse. CYP27B1 and CYP24A1 transcripts are expressed to similar levels in the kidney of the horse, whereas in the dog and the sheep CYP24A1 expression was higher than that of CYP27B1 [119].

Few studies have analyzed 25(OH)D2 and 25(OH)D3 in horse serum using a technique that can differentiate the two metabolites [110,112,113,120,121]. A recent study that evaluated serum 25(OH)D levels in horses using LC-MS/MS (which does distinguish between 25(OH)D2 and 25(OH)D3) demonstrated that 25(OH)D2 was the predominate 25(OH)D metabolite in horses [88]. 25(OH)D3 was undetectable above the lower limit of quantification (which was not specified) and there was no difference between horses that were blanketed or unblanketed for the study period of 13 months [88]. This result was unexpected given that skin coverage has a direct impact on 25(OH)D3 synthesis in other mammalian skin [122,123,124]. Interestingly however, serum 25(OH)D2 concentrations followed a seasonal pattern, decreasing in the winter months with the highest concentrations detected in the summer (again with no difference detected between blanketed and unblanketed animals) [88]. The same seasonal pattern was also determined for 25(OH)D2 measured in the grazing pasture consumed by the horses [88]. These results suggest that horses may rely on vitamin D2 from foodstuff to fulfill their vitamin D requirements. The horses in the study discussed here all had dark skin [88], and given the role of pigmentation in cutaneous vitamin D production [125] further investigation into the cutaneous production of vitamin D3 in horses is warranted. Also of note, vitamin D metabolites have been shown to exhibit both seasonal (25(OH)D2) and circadian (25(OH)D3 and 1,25(OH)_2_D) fluctuations in horses [88,121,126], as well as being influenced by exercise (vitamin D3 but specific metabolite not specified) [127]. To the authors knowledge, C3-epimers of vitamin D metabolites have not been measured in horses.

Although naturally occurring rickets is rare in horses, Shetland ponies deprived of sunlight and dietary vitamin D can develop early signs of rickets [110]. Conversely, during vitamin D intoxication the typical increase in serum calcium and 1,25(OH)_2_D concentrations is much reduced in comparison with other species, yet there is a marked increase in response of inorganic phosphate [128]. This led Harmeyer et al. (2004) to demonstrate that preventing the formation of a calcium-inorganic phosphate product by reducing the content of these in food can reduce soft tissue calcification that occurs in horses due to vitamin D intoxication [128].

Studies investigating the extraskeletal effects of vitamin D in the horse are still scarce; however, there is evidence of a relationship between vitamin D status and equine perinatal diseases. The concentration of vitamin D in healthy foals is lower than the already low concentrations detected in adult horses; and in hospitalised foals (a mixture of sepsis and non-sepsis patients), low 25(OH)D3 and 1,25(OH)_2_D3 concentrations in comparison to healthy foals correlates with disease severity and mortality [129]. The authors observed that vitamin D deficiency combined with hypocalcemia, hyperphosphatemia, and high PTH concentrations in septic foals may point to PTH resistance being associated with the development of these abnormalities [129]. The group goes on further to identify FGF23/klotho imbalances as contributors to disease progression in the equine neonate [130].

#### 3.1.3. Sheep and Goats

There are mixed reports regarding the major form of vitamin D in sheep. Recent studies on farmed flocks in Scotland and New Zealand have demonstrated significant contribution from both D3 (cutaneous origin) and D2 (dietary origin) [90,91], however a study on an unmanaged population of Soay sheep on the island of St Kilda reported D3 as the major contributor [30]. Given that D2 is solely of dietary origin, this may be reflective of the quality of pasture available to intensively farmed sheep in comparison to wild Soay sheep on the St Kilda islands. Growing lambs and kids have been shown to be able to compensate for reduced dietary vitamin D intake by cutaneous production [131]. Interestingly, 25(OH)D3 concentrations were substantially greater in the New Zealand flock [90] in comparison to both the Scottish Blackface [91] and Soay flocks [30]. This is likely the result of breed skin pigmentation differences, with the Romney sheep of New Zealand having minimal skin pigmentation in comparison to the Soay sheep and the Scottish Blackface sheep which both have dark skin and significant pigmentation. Comparing the Scottish breeds in the study by Zhou et al., the concentration of 25(OH)D3 and total 25(OH)D was significantly higher in Lleyn ewes, which have light pigmentation and white faces and legs, in comparison with Scottish Blackface ewes which have dark pigmentation and black faces and legs [91]. Combined, these results support the findings from Handel et al. (2016) that sheep coat colour impacts vitamin D3 status [30]. It has been demonstrated that a heavy fleece and pigmented skin reduce cutaneous vitamin D biosynthesis in comparison to shorn sheep with white face and legs [132]. Combined with reduced UVB radiation in late winter, this leads to a seasonal trough in serum 25(OH)D concentration in temperate regions which can be exacerbated by the demands of pregnancy [133,134]. Generally, rickets due to vitamin D deficiency is uncommon in sheep; however, UV radiation is only adequate for the synthesis of vitamin D between mid-March and mid-September at latitudes greater than 55° N; regions at or above these latitudes have reported instances of rickets of nutritional origin [13].

Unlike monogastric animals, small ruminants do not modulate renal calcium excretion in response to dietary calcium restriction. Goats have been shown to have a greater capacity to compensate for challenges of calcium homeostasis compared to sheep [135]. In comparison to goats, sheep are more dependent on dietary intake of vitamin D, with the concentration of 7DHC in the skin of sheep shown to be less than 10 times that in the goat [136], potentially explaining the decreased incidence of rickets in goats [135]. Interestingly, a genetic mutation in Corriedale sheep in New Zealand has recently been identified with similarities to autosomal recessive hypophosphatemic rickets (VDDR type 2) in humans [137,138,139].

Of particular interest is the relationship between vitamin D status and reproductive fitness in sheep. Gestation and lambing commonly coincide with an ewe’s seasonal decline in vitamin D status [140]. Supplementing pregnant ewes with vitamin D has shown to improve the vitamin D status of lambs, however lamb vitamin D status still remains much lower than that of the ewe [134]. Recently, novel associations between vitamin D status and reproductive fitness in sheep have been identified. In an unmanaged wild population of Soay sheep on the island of St Kilda, vitamin D status was demonstrated to be both heritable and under natural selection [30]. Total 25(OH)D serum concentration of the ewe was positively associated with the number of lambs that survived for one-year [30]. Subsequently, a study by Zhou et al. (2019) examined reproductive traits and vitamin D status in Scottish hill sheep. This study observed no significant association between ewe vitamin D status and number of lambs born or weaned, however concentrations of 25(OH)D3 and total 25(OH)D were positively associated with birth weight of single and twin lambs [91]. Conversely, a recent study on a flock of Romney sheep in New Zealand revealed a negative correlation between total 25(OH)D concentration and ewes that were pregnant with triplets, with no association detected between vitamin D status and ewes pregnant with single or twin lambs [90]. The mean total 25(OH)D concentrations of the Romney sheep in New Zealand was substantially higher than that of the Scottish sheep reported by Zhou et al. (2019) (97.91 nmol/L in Romney sheep versus 36–45 nmol/L in Scottish sheep), leading the author to postulate that the positive effect of increasing serum 25(OH)D concentrations on fecundity may only occur when vitamin D concentrations are marginal [90], as seen in both the Scottish sheep [91] and Soay sheep [30]. A recent preliminary study has also reported reduced instances of vaginal prolapse in pregnant ewes administered with injectable vitamins A, D3 and E, which warrants further investigation [89].

Vitamin D has also been implicated in spermatogenesis and sperm maturation in sheep [141]. In the male sheep reproductive tract, the VDR, CYP24A1 and CYP27B1 have been shown to be differentially expressed at different developmental stages and in different sources of spermatozoa [141]. The expression patterns of the VDR and vitamin D enzymes suggest potential for modulating a local vitamin D response in the reproductive organs, and high VDR and CYP24A1 expression in high-motility spermatozoa suggests that sperm activity may require vitamin D under tight regulation [141]. Given the potential for using an ovine model of vitamin D metabolism in pregnancy [142], further investigation surrounding the role of vitamin D and reproductive fitness in this species is warranted.

#### 3.1.4. Cattle

Supplementation of both dairy and beef cows with vitamin D is recommended. The impact of farming practices and potential limited sun exposure, combined with increased metabolic demands of the periparturient period, advocates that cows should be supplemented with vitamin D to maintain calcium homeostasis and immune system function. Seasonal variation in vitamin D status is well accepted in cattle in temperate regions [143,144,145]. Investigations into the variation in vitamin D status throughout different stages of lactation demonstrated a depletion of 25(OH)D in dairy cows postpartum versus late prepartum [146] and it is known that calves, as with newborns of other species, have lower serum 25(OH)D than adult cows.

There is interest in increasing the vitamin D content in both milk and meat in order to benefit the consumer of beef and dairy products. As such, studies investigating the most efficient way to supplement cattle with vitamin D have been conducted, comparing whether vitamin D or 25(OH)D supplementation is most effective. Supplementation with 25(OH)D3 over vitamin D3 has been demonstrated to improve plasma concentrations of 25(OH)D more effectively [92,93]; there was no difference between the two metabolites ability to improve milk 25(OH)D concentrations [93] but 25(OH)D supplementation increased 25(OH)D3 levels in tissues greater than supplementation with vitamin D3 [92]. Weir et al. (2017) have reviewed environmental and genetic factors that influence the vitamin D content of cow’s milk [145]. Factors such as UVB exposure, diet, farming practices (in particular the impact of year-round housing), breed, hair colour, age and stage of lactation were identified, warranting further investigation to fully elucidate how farmers could manipulate these factors to increase vitamin D content of milk [145].

As with other species, the skeletal benefits of maintaining sufficient vitamin D status are well accepted. Although the extra-skeletal effects of vitamin D are less well studied in ruminants, vitamin D metabolites have been shown to modulate bovine immune cells both in vitro and in vivo [147,148,149,150,151,152]. Studies investigating associations between vitamin D status and infectious disease in cattle have, however, produced conflicting results [153,154].

#### 3.1.5. Pigs

Pigs are particularly sensitive to developing conditions related to vitamin D deficiency, such as rickets and fibrous osteodystrophy, as a result of rapid growth rates and early weaning. Rapid growth and early weaning, combined with the controlled indoor environment in many modern intensive pig farming facilities that often have restricted sunlight, mean that adequate supplies of dietary vitamin D are a necessity [155]. Recent studies have demonstrated that outdoor sun exposure does increase serum 25(OH)D concentrations in growing pigs and this can be more effective at increasing serum 25(OH)D than dietary supplementation [94,156]. Flohr et al. (2016) demonstrated that dietary supplementation with 25(OH)D3 was more efficient than vitamin D3 supplementation at increasing serum 25(OH)D concentrations in sows, and resulted in faster growing piglets [157,158].

A genetic form of rickets, pseudo-vitamin D dependent rickets type 1 (PDDR1) has been characterized in Hannover pigs and was utilized as a model for vitamin D dependent rickets type 1 (VDDR1) in humans. The condition is a result of coding-region deletions in CYP27B1 which renders the enzyme ineffective, meaning that these animals have an inability to maintain ambient levels of 1,25(OH)_2_D [159,160,161].

#### 3.1.6. Poultry

Vitamin D deficiency in chickens can result in economic loss due to its dual actions in calcium absorption and bone mineralization in fast growing birds raised for meat [162,163] and in its essential role in egg shell calcification and decalcification (for embryonic bone formation and hatching) in laying hens [78,164,165]. Birds, such as chickens, that lay hard-shelled eggs require rapid calcium supply to the uterus for calcium deposition. Vitamin D is highly involved in both intestinal and uterine calcium transport, therefore 1,25(OH)_2_D markedly fluctuates with the ovulatory cycle of birds [166,167]. Consequently, dietary supplementation of vitamin D is vital and supplementation with 25(OH)D3 has shown some benefits over supplementation with vitamin D3 [168], including increased cellular immune response, improved mineral deposition in bones of broilers [169] and improved sternum structure and mineral accretion [170]. Hutton et al. (2014) have demonstrated that supplementation with 25(OH)D3 improves breast meat yield in broilers by stimulating skeletal muscle satellite cells [171]. Supplementation with both vitamin D3 and 25(OH)D3 has been shown to increase the vitamin D content of egg yolks, a potential fortification method for human consumption [172]. Supplementation with D3 metabolites rather than D2 is more effective in increasing vitamin D content of egg yolks [173], possibly due to 25(OH)D2 being less well bound to the VDBP than 25(OH)D3 in chickens, rendering it less effective [174].

Vitamin D3 can be cutaneously produced in chickens, however the anatomical location and the presence of feathers can impact this process [95,96]. The concentration of 7DHC in the skin of chickens (the limiting factor in the cutaneous production of vitamin D3) is variable across anatomical locations [95,96]. Schutkowski et al. (2013) demonstrated that the highest concentration of 7DHC is found in the skin of unfeathered legs of chickens, with the comb and wattle containing significantly (190-fold) lower concentrations; feathered legs and wings had the lowest concentrations of 7DHC [96]. Kuhn et al. (2015) later demonstrated similar results, showing that feathered leg skin contains lower concentrations of 7DHC than unfeathered leg skin; and exposure of the skin to UVB radiation resulted in a significant increase in the concentration of vitamin D3 and 25(OH)D3 in unfeathered skin only [95]. These results had important implications on future studies that investigated the exposure of chickens to UVB radiation (in an attempt to increase vitamin D status and content of egg yolks), as they revealed the importance of UVB lamp placement. Subsequently, exposure of birds to UVB has been shown to be effective at increasing vitamin D content in eggs in a non-linear fashion [95], with both exposure to natural sunlight via free range farming practices [175] and artificial light regimes for indoor caged birds being effective [176]. Interestingly, an older study by Lietzow et al. (2012) conversely reported no benefit of short-term UVB exposure of laying hens to improve vitamin D content of egg yolks [177]. However, in the study by Lietzow et al., the UVB lamps were placed above the hen’s heads, which is now known to not be the optimal position due to the highest concentration of 7DHC being present in the unfeathered legs.

Some avian species have a uropygial gland (often referred to as the oil or preen gland), cranial to the implanting tail feathers, that can be involved in vitamin D synthesis [178,179,180,181,182]. The gland secretes oil that contains a complex mixture of ester waxes, fatty acids, lipids and wax alcohols, and its composition is species dependent [179,180,181,182]. The oil is spread among the plumage at preening, with some of the main functions including antimicrobial activity, anti-abrasive effects, hydrophobic properties (for water-proofing), production of pheromones and sex linked changes [179]. In some species, the oil contains 7DHC which, after spreading over the feathers, can be exposed to UVB radiation. This results in vitamin D3 synthesis that can subsequently taken up by the bird during further preening [178,179]. There is currently limited research into whether this vitamin D intake occurs in chickens and if so, the contribution of this vitamin D3 synthesis to vitamin D status is still unknown.

#### 3.1.7. Llamas and Alpacas

Llamas and alpacas are evolutionarily adapted to high altitude environments with high levels of solar radiation, and rely heavily on cutaneous production of vitamin D. When moved to other climates, particularly to temperate regions, these species become more susceptible to rickets [77,183]. Seasonal variation in vitamin D status has been demonstrated in both of these species [184] and it is recognized that crias born in the autumn and winter months have lower vitamin D status and therefore higher risk of developing rickets than those born in the summer [185]. Interestingly, like sheep, alpacas with dark coats have a lower vitamin D status than those with light coats in late winter [186]; however, alpacas seem to be more susceptible to rickets than sheep, with alpacas becoming hypophosphatemic and developing rickets during winter months in New Zealand whereas lambs grazing on the same pasture showed no signs of deficiency [187]. This is likely reflective of the high reliance of alpacas on cutaneous vitamin D production.

#### 3.1.8. Nonhuman Primates

Nonhuman primates have been shown to exhibit much higher serum 25(OH)D3 and 1,25(OH)_2_D3 concentrations than humans [97] (Figure 2A). Marmosets in particular have very high 25(OH)D and 1,25(OH)_2_D3 levels [97,188,189]. These high levels of 1,25(OH)_2_D3 are suggestive of end-organ resistance [189,190] and have been demonstrated to be caused by the overexpression of a VDR-independent VDRE-binding protein which interferes with vitamin D-regulated transactivation [191], making this species a useful model for hereditary vitamin D resistant rickets (HVDRR). Interestingly, striking levels of variation in vitamin D status within species was detected even in laboratory primates that were subject to controlled diet and UV exposure [97]. Additionally, vitamin D status has been demonstrated to be lower in darker skinned baboons than in those with lighter skin, however there were no differences in downstream metabolites suggesting that downstream conversion is under strong regulatory control [98].

### 3.2. Profiling Vitamin D beyond 25-Hydoxyvitamin-D in Veterinary Species

#### 3.2.1. Measuring 1α,25-Dihydroxyvitamin-D

As in humans, the clinical value of directly measuring 1,25(OH)_2_D in specific disease groups is being realised in veterinary medicine. Although uncommon, there are several disorders of disturbed vitamin D metabolism in which 1,25(OH)_2_D concentrations may be increased or decreased to undesirable levels and may not always be reflected by altered 25(OH)D levels [192]. These conditions can be grouped into distinct origins of 1,25(OH)_2_D disturbance; 1α-hydroxylase deficiencies, mutations of the VDR, and excessive extrarenal production.

1α-hydroxylase deficiencies include vitamin D dependent rickets type 1, an autosomal recessive disorder causing an inactivating mutation in CYP27B1, the 1,25(OH)_2_D producing enzyme. This is a rare disease that causes abnormally low concentrations of 1,25(OH)_2_D and the early onset of rickets [193]. Congenital disorders of vitamin D metabolism are rare in animals, but several cases have been reported in a range of species. Vitamin D dependent rickets (VDDR) type 1A, in which CYP27B1 contains an inactivating mutation resulting in reduced conversion of 25(OH)D to 1,25(OH)_2_D, has been reported in cats [194,195], an unconfirmed case in a dog [196] and in Hannover pigs [159,161]. In the case of the cats and the pigs, serum 25(OH)D were normal to high, however 1,25(OH)_2_D concentration was low; vitamin D metabolites were not measured in the case of the dog. More recently, a case of VDDR type 1B was described in a cat for the first time [197]. Here, a frameshift mutation at exon 5 in CYP2R1 was identified, resulting in an inability to convert vitamin D into 25(OH)D [197]. Both disorders can be managed by supplementation with 1,25(OH)_2_D, with varying degrees of success.

The second origin of 1,25(OH)_2_D unbalance are those disorders exhibiting mutations of the VDR. Mutations resulting in the VDR becoming unresponsive or less responsive to its substrate include hereditary vitamin D resistant rickets (vitamin D dependent rickets type 2) and result in hypocalcaemia and early onset rickets. In these patients, very high circulating concentrations of 1,25(OH)_2_D are recorded [198]. A few cases of VDDR type 2, involving mutations of the VDR gene, have been reported in animals. In the case of two cats [199,200] and a dog [201], the animals presented with clinical signs of early onset rickets, hypocalcaemia, secondary hyperparathyroidism and increased concentration of 1,25(OH)_2_D. Treatment of this condition is challenging and consists of high doses of calcium and 1,25(OH)_2_D. Corriedale sheep with features of VDDR type 2 have also been reported [137,138]. These animals presented clinical signs of rickets, significant hypocalcaemia and hypophosphatemia, normal serum 25(OH)D but high serum 1,25(OH)_2_D concentrations, suggesting end organ resistance to 1,25(OH)_2_D typical of VDDR type 2 [137]. However, subsequent in vitro studies revealed that cultured skin fibroblasts from affected animals exhibited normal VDR function and demonstrated an increase in CYP24 mRNA expression, suggesting that 24-hydroxylase may be involved in the pathogenesis [202]. Furthermore, mutations in the dentin matrix protein 1 gene (DMP1) were identified in affected animals, and are known to be involved in autosomal recessive hypophosphatemic rickets humans [139]. These results suggest that the genetic defect in the Corriedale sheep may be different to the genetic defects described in other species with VDDR type 2 to date. A DMP1-knockout mouse model of autosomal recessive hypophosphatemic rickets exists and exhibits inappropriately normal serum 1,25(OH)_2_D in response to elevated FGF23 [203,204], and there are four strains of VDR null mice which are phenotypically similar to VDDR type 2 in humans [205].

Excessive extra renal production of 1,25(OH)_2_D can also occur in animals. Typically, this occurs in patients with granulomatous diseases, where a dysregulated immune response results in the excessive production of 1,25(OH)_2_D, typically by macrophages. This syndrome has been reported in dogs with sterile granulomatous lymphadenitis [206], granulomatous inflammation following placement of a biological implant [207], *Angiostrongylus vasorum* infections [208], *Mycobacterium avium subspecies hominissuis* infection [209], and blastomycosis infection in a cat [210]. Excessive production of 1,25(OH)_2_D has also been postulated to be important in driving hypercalcaemia in dogs with autoimmune diseases such as immune mediated polyarthritis [211]. Successful treatment of the underlying condition typically resolves the increase in systemic 1,25(OH)_2_D concentrations and associated hypercalcaemic state.

Low concentrations of 1,25(OH)_2_D have been reported in numerous health conditions in companion animals. In cats and dogs with chronic kidney disease, low 25(OH)D concentration and reduced CYP27B1 activity due to elevated levels of FGF23, contribute to low circulating 1,25(OH)_2_D concentrations [25,212]. The decline in 1,25(OH)_2_D concentrations is considered important in the development of secondary hyperparathyroidism in chronic renal disease, leading to interest in the potential therapeutic merits of 1,25(OH)_2_D supplementation in companion animals with renal failure [212,213,214]. In canine cancer patients, measuring serum 1,25(OH)_2_D concentrations in dogs with lymphoma with and without hypercalcaemia has produced variable results [12,215,216]. Dogs with protein losing enteropathy have significantly lower serum concentrations of 1,25(OH)_2_D compared to dogs with a chronic enteropathy and normal albumin concentrations or healthy dogs [22,23]. In cases of canine primary hyperparathyroidism, 1,25(OH)_2_D is significantly increased in comparison to healthy dogs due to upregulation of 1α-hydroxylase by the action of PTH [216].

#### 3.2.2. 24,25-Dihydroxyvitamin D and Vitamin D Metabolite Ratios

The assessment of 24,25(OH)_2_D is reported infrequently in the veterinary literature. Although mutations in CYP24A1 have not been reported in animals, studies are beginning to reveal potential associations between 24,25(OH)_2_D concentration and health and disease, particularly in dogs. Tryfonidou et al. (2002) described 24-hydroxylase as a key regulator in hypervitaminosis D in growing dogs [217] and subsequently observed differences in vitamin D metabolite concentrations between large and small breed dogs; 1,25(OH)_2_D was increased and both 25(OH)D and 24,25(OH)_2_D concentrations decreased in Great Danes in comparison to Miniature Poodles [218].

A study investigating the relationship between exercise and vitamin D metabolism in racing sled dogs demonstrated that, contrary to human athletes, 25(OH)D concentration increased by day 2 of exercise and a subsequent significant increase in 24,25(OH)_2_D concentration by day 8 was detected. This led the author to postulate a possible homeostatic mechanism whereby 24-hydroxylase activity was increased in order to decrease 25(OH)D concentration [87].

In dogs with stage 3 and 4 CKD, 24,25(OH)_2_D concentrations were significantly lower than in control dogs or those with stage 1 and 2 disease [25]. Both of these studies reported higher concentrations of 24,25(OH)_2_D in dogs in comparison to other species, suggesting enhanced 24-hydroxylase activity as a result of higher vitamin D intake [25,87]. Dogs fed AAFCO or FEDIAF approved diets tend to have higher serum concentrations of 25(OH)D than humans which supports the hypothesis that dogs may have increased 24-hydroxyalse activity and therefore 24,25(OH)_2_D concentrations to regulate this increased intake. Young et al. (2017) have also demonstrated that supplementation with 25(OH)D3 significantly increases 24,25(OH)_2_D concentration, further supporting this hypothesis [219]. These studies highlight the need for further investigation into the relationship between vitamin D intake and status in dogs, particularly as there appears to be some metabolic variation in this species. Given that genome wide studies have identified CYP24A1 as a genetic determinant of 25(OH)D status [220], understanding vitamin D catabolic activity may also reveal insights into why 25(OH)D status may differ in otherwise similar populations. For example, in a longitudinal study of 18 dogs on a standardized diet, variation was detected between individual animals [80] suggesting host factors play a major role in regulating and maintaining vitamin D status, of which CYP24A1 activity may be one. In a study investigating vitamin D in an unmanaged Soay sheep population, some animals have consistently higher concentrations of 25(OH)D in comparison to others [30]. Although coat colour was identified as a determinant of vitamin D status in these animals, it did not explain all the variation and further profiling of the vitamin D metabolites and their enzymes could provide key information as to why this variation exists.

The assessment of vitamin D metabolite ratios (VMRs) in animals was described as early as 1982 by Horst et al. in a study comparing vitamin D metabolites in domestic species [221]. They describe sheep and pigs to have a higher ratio of 24,25(OH)_2_D:25(OH)D than turkeys and chickens; and in cows, although inorganic phosphate was high and calcium was normal as in sheep and pigs, they had a similar 24,25(OH)_2_D:25(OH)D ratio to turkeys and chickens, suggesting reduced efficiency of conversion of 25(OH)D to 24,25(OH)_2_D [221]. However, the assessment of VMR remains less commonplace in animals than in humans. Recently, Groth et al. (2019) examined both the 25(OH)D:24,25(OH)_2_D and 1,25(OH)_2_D:25(OH)D ratios, as well as individual metabolite concentrations in dogs with and without hypercalciuric calcium oxalate urolithiasis [82]. Interestingly, none of the individual metabolite measurements (25(OH)D, 1,25(OH)_2_D and 24,25(OH)_2_D) yielded significant differences between cases and the control group, neither did the 1,25(OH)_2_D:25(OH)D ratio; however, the 25(OH)D;24,25(OH)_2_D ratio was significantly higher in cases versus controls [82]. Although ranges overlapped, 6 out of 19 cases had ratios higher than the highest ratio observed in the control group, with the author postulating that decreased 24-hydroxylase activity on 25(OH)D might contribute to calcium oxalate urolithiasis in some but not all dogs. Further investigation is necessary to determine whether this change is representative of disease in the case group or a preventative mechanism in the control group [82]. Additionally, of note, was the variability in the 25(OH)D;24,25(OH)_2_D ratio, not only between breeds but within breeds [204]. Once again, this highlights the need to further understand the role of host factors in vitamin D metabolism.

Assessing VMR’s in the study by Groth et al. (2019) increased the power of detection of differences in vitamin D status between populations in comparison to comparing individual metabolites [82]. Considering the narrow range of 24,25(OH)_2_D:25(OH)D ratio in vitamin D sufficient people, assessing VMR’s in animals could provide useful information to further clarify vitamin D status and improve the power of detecting differences between populations. Given the evidence regarding increased activity of CYP24A1 in dogs, measuring VMR’s could also impact the way in which vitamin D metabolites are used as treatments in animals. The 24-hydroxylase enzyme can also act upon 1,25(OH)_2_D, meaning conditions in which 1,25(OH)_2_D is used as treatment (such as 1α-hydroxylase deficiencies) could also benefit from this information and begin to address the variability in response to treatment. In humans, a significant increase in 1,25(OH)_2_D:24,25(OH)_2_D VMR was detected during vitamin D insufficiency, suggesting 24-hydroxyalse activity is partially inactivated in order to maintain adequate substrate for 1α-hydroxylation, and implying that 24-hydroxylase activity may be increased in order to convert excess 25(OH)D into 24,25(OH)_2_D in cases of hypervitaminosis D [222]. Again, given the potential variation in vitamin D metabolism regarding CYP24A1 activity in dogs, and the potential differences in tolerance of high levels of vitamin D in cats, it would be of benefit to examine these metabolites in these species.

#### 3.2.3. C3-Epimers

The 3-epi-25(OH)D metabolite has now been detected in both cats [85] and dogs [81] with mean concentrations reported as 58.7 nmol/L (23.5 ng/mL) and 5.2 nmol/L (2.1 ng/mL) respectively (Figure 2B). There have not been any studies investigating the relationship between the C3-epimers and health and disease in animals. In humans, concentrations of the C3-epimers are increased during pregnancy [223] and concentrations are higher in babies than in adults (Figure 2B). It would be of interest to determine whether the same pattern is detected in animals by measuring 3-epi-25(OH)D in newborns. Of particular interest would be the change in C3-epimer concentrations in both the mother and young before, during and after weaning onto a commercial diet with known amounts of vitamin D; as in human premature babies, 3-epi-25(OH)D increased significantly with supplementation in comparison to cord blood measurements [224].

In dogs, variation in 3-epi-25(OH)D was detected between animals [81] and factors contributing to this variation should be investigated. Given that dogs demonstrate similar levels of 3-epi-25(OH)D relative to their total 25(OH)D concentration as humans, combined with the ease of controlling vitamin D intake through consumption, it would be useful to model the C3-epimerization pathway during pregnancy in this species. Ultimately, given the potential for C3-epimers to interfere with other vitamin D metabolite analysis, the biological activity and role of the C3-epimers needs to be well defined in animals and the concentrations of these metabolites in various healthy and disease groups should be determined.

#### 3.2.4. Free Vitamin D and the Vitamin D Binding Protein

The recent interest in analyzing free 25(OH)D has yet to be reflected in the veterinary field. The free hormone theory states that only the free (unbound) molecules can passively diffuse across cell membranes and therefore be biologically available [225,226,227]; however, several target organs of 25(OH)D express the transmembrane protein megalin, which can mediate internalization of VDBP-bound metabolites [227,228,229]. The importance of free 25(OH)D for biological functions and the role of VDBP as a reservoir has been demonstrated by mice lacking the VDBP. These animals presented with markedly reduced circulating levels of 25(OH)D, however did not develop rickets until placed on a low vitamin D diet [230]. Current interest surrounding the measurement of free 25(OH)D stems from the consideration that free 25(OH)D may better represent vitamin D status in some populations; in particular those with physiological or pathological conditions in which VDBP is altered [227]. The utility of free 25(OH)D as a biomarker will, however, be dependent upon concentrations or correlations of free 25(OH)D and total 25(OH)D being divergent, in order to provide information not already provided by measuring total 25(OH)D [231]. In healthy human populations, significant correlations between free 25(OH)D and total 25(OH)D have been demonstrated [3,227,232]. However, free 25(OH)D concentrations or the relationship between free and total 25(OH)D may be altered in clinical conditions in which VDBP is altered (a number of medications, hormones and smoking have been shown to affect VDBP levels [233]), the affinity of vitamin D metabolites to VDBP or albumin is altered (for example, different VDBP haplotypes), or the disposition of vitamin D itself is disturbed.

Very limited studies (especially in recent times) have examined species-based differences in VDBP levels, or primary protein sequences of the VDBP (as they relate to affinity of vitamin D metabolites) between species [234,235,236,237,238,239,240]. Circulating vitamin D metabolite concentrations are dependent on affinity to the VDBP, as well as metabolism and dietary intake [241,242,243]. Therefore, species differences in the VDBP may rationalize species-based differences in circulating vitamin D metabolite levels. Studies are also limited in the analysis of free 25(OH)D in animal species, with only one study to date reporting directly measured concentrations of this vitamin D metabolite in animals. Hurst et al. (2020) reported for the first time that free 25(OH)D can be measured in canine serum, with a median concentration of 15.2 nmol/L (6.1 ng/mL) in a group of 117 healthy dogs [81]. In this study, free 25(OH)D was measured by the Free 25(OH)D ELISA (FutureDiagnostics) and was positively correlated to both 25(OH)D3 and 3-epi-25(OH)D3 concentrations [81]. The concentrations reported in dogs are comparable to free 25(OH)D concentrations reported in humans [244], and importantly, the relative percentage of free 25(OH)D to total 25(OH)D is also comparable (0.02% in dogs and 0.03% in healthy humans as reported by Bikle et al. [244]). Hurst et al. (2020) reported that no correlation was detected between either free 25(OH)D or total 25(OH)D with PTH in dogs.

## 4. Analytical Challenges in Profiling Vitamin D Metabolites in Veterinary Species

**Figure 3:**
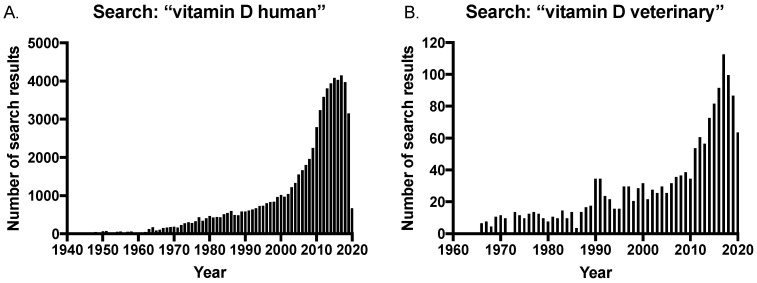
Number of search results in PubMed^®^ for searches on (**A**) vitamin D human and (**B**) vitamin D veterinary, over time.

The long-established importance of maintaining vitamin D sufficiency has been further corroborated given the discovery of extra-skeletal effects of vitamin D, and this has led to an increase in studies investigating vitamin D levels and disease in both humans and veterinary species. This is reflected in the growing number of publications on the subject, with a rapid increase in the number of search results on PubMed^®^ for vitamin D and humans in the last 20 years (Figure 3A). In contrast, there was a delay in the veterinary field with an increase in publications only occurring within the last 10 years (Figure 3B); and significantly, the number of search results for “vitamin D veterinary” is approximately only 2.5% of that of the human search results at the peak of publications in 2017 (searched on pubmed.ncbi.nih.gov in July 2020).

Nonetheless, the increased interest in vitamin D in human medicine has led to increasing demand for the measurement of vitamin D metabolites in veterinary species, both as a clinical diagnostic and research tool. There are a number of analytical techniques for the quantification of vitamin D metabolites, including enzyme-linked immunoassays (ELISA), radioimmunoassay (RIAs), chemiluminescence assays, HPLC, HPLC combined with UV detection and uHPLC or HPLC combined with tandem mass spectrometry (LC-MS/MS) [245]. Quantification of vitamin D metabolites, even the main circulating metabolite 25(OH)D that is used routinely to determine vitamin D status, is challenging. The vitamin D metabolism pathway is a highly complex and dynamic system involving a number of structurally similar compounds that can cause interference with analysis; not only that, the metabolites circulate predominantly bound to the VDBP and at low concentrations. All of these factors mean that vitamin D metabolite measurement is particularly challenging. Discussed in detail below is how LC-MS/MS is well suited to address many of these issues and, just as in analysis of sex steroids [246], is considered the gold-standard analytical technique for vitamin D measurement [245,247].

Although there is increased demand for vitamin D testing in veterinary patients, the field is still behind human vitamin D clinical and research studies in terms of the application of the gold-standard method for assessing vitamin D. This was highlighted after a search of the cited literature in this review, combined with searching PubMed^®^ for keywords “vitamin D veterinary LC-MS/MS”. Table 1 shows the results of these searches and is a non-exhaustive list of publications which have measured vitamin D metabolites in veterinary species by LC-MS/MS in the past 10 years, comparing method details when available. Of the searched literature, there were fewer than 30 publications noted to use LC-MS/MS for vitamin D analysis, or that acknowledge LC-MS/MS in the publication’s keywords. Notably, of the few publications outlined in Table 1 that did use LC-MS/MS for veterinary vitamin D analysis, more than half failed to provide details for 2 or more LC-MS/MS method parameters listed in the table; with many simply stating that samples were analysed by an external laboratory without providing any details of the method. These method details are important for analytical comparisons. Many published veterinary case studies in which vitamin D measurements have been used for diagnostic purposes do not specify how vitamin D metabolites were measured. Research publications in veterinary species typically use either immunoassay-based techniques, HPLC alone or coupled with UV detection. The lack of reporting of methods and the various techniques used for vitamin D analysis, combined with the lack of transparency of analytical method details, certainly emphasizes that caution must be used when comparing study results due to lack of standardization. Figure 2 displays data for the vitamin D metabolite concentration ranges measured by LC-MS/MS in healthy adult populations of species from the studies outlined in Table 1.

There are many extensive reviews comparing and discussing the different methods for the quantification of vitamin D metabolites, with many focusing particularly on LC-MS/MS methods (in humans) and the particular challenges of quantifying each metabolite in detail [33,248,249,250,251,252,253]. The author directs the reader to this referenced literature for detailed comparisons of reported vitamin D LC-MS/MS methods. The next section of this review will focus on the analytical challenges of quantifying vitamin D metabolites in veterinary species.

### 4.1. Analytical Challenges of Vitamin D Analysis

Many clinical laboratories and research studies use immunoassay-based techniques, at least for the initial assessment of 25(OH)D and 1,25(OH)_2_D3. These assays are provided in kits and are easily integrated into fully-automated laboratory systems allowing for rapid analysis in a high-throughput clinical chemistry laboratory setting. They offer good sensitivity and require minimal sample volume. However, they are restricted in the analytes they can measure, not only requiring different methods or kits for 25(OH)D and 1,25(OH)_2_D, but other metabolites which may be of interest (for example, the C3-epimers and 24,25(OH)_2_D3) are not available. Lack of specificity for the analytes they can measure continues to be one of their major limitations [251,252,253]. Cross reactivity with different vitamin D metabolites occurs in many of the immunoassays. Lack of selectivity between 25(OH)D2 and 25(OH)D3, and unequal cross reactivity of the two metabolites can cause bias and have a significant impact depending on the sample being analyzed. Other metabolites such as 24,25(OH)_2_D have been demonstrated to cross react to varying degrees in immunoassays from different vendors [251]. Although 24,25(OH)_2_D3 generally circulates at a lower concentration than 25(OH)D3 in humans, clinical situations in which 24,25(OH)_2_D is increased, or in species with higher baseline concentrations of 24,25(OH)_2_D, this interference could overestimate 25(OH)D quantification and result in miss-classification. Furthermore, vitamin D metabolites must be released from the VDBP in order to be measured, which is difficult to achieve in automated immunoassays in which strong organic solvents cannot be used [251,252]. Therefore, samples in which variation in the VDBP levels exists (during pregnancy or cases of renal disease for example) are known to impact on the performance of these assays.

**Figure 4:**
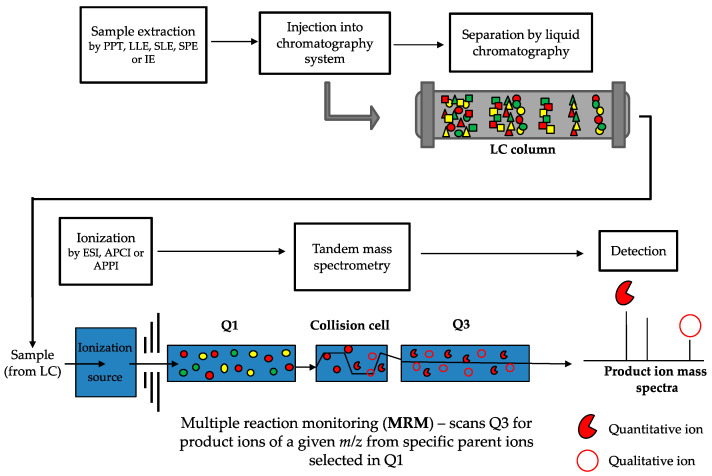
Workflow of LC-MS/MS method for vitamin D metabolite quantification. Samples (usually serum) are prepared by an extraction method (such as protein precipitation (PPT), liquid-liquid extraction (LLE), supported liquid extraction (SLE), solid phase extraction (SPE) or immunoextraction (IE)) and then injected into the liquid chromatography system. Metabolites are chromatographically separated based on physical and chemical interactions (represented by the different shapes) with the LC column and are introduced to the ionization source of the mass spectrometer. Metabolites are ionized by either electrospray ionization (ESI), atmospheric pressure chemical ionization (APCI), or atmospheric pressure photoionization (APPI). During tandem mass spectrometry, when multiple reaction monitoring (MRM, which is the same as selected reaction monitoring but more than one reaction is monitored) is used, a predefined parent ion is identified based on its *m/z* transition (the full red circles, with other colours representing different parent ions) in the first quadrupole (Q1) and is selected to enter the collision cell. In the collision cell, the parent ion is fragmented into defined product ions, which are then passed through into the third quadrupole (Q3). In Q3, defined quantitative and qualitative product ions (the red pie and open circle, respectively) are detected and used for identification and quantification. The peak response of the quantitative and qualitative product ions is converted into a ratio which should be consistent across samples and standards. Deviation from this ratio of more than 15% to 20% can indicate potential interference.

LC-MS/MS is considered the gold-standard technique for analyzing vitamin D metabolites [245,247]. This method addresses the limitations outlined above for immunoassays. There are a number of steps involved in LC-MS/MS methods (a typical workflow is outlined in Figure 4), all of which play an important role in the accurate and precise quantitation of vitamin D metabolites. Samples are initially prepared in order to clean up and eliminate any potential interfering compounds from the biological matrix, and to concentrate the molecules of interest. Analytes are then chromatographically separated based on physical and chemical interactions with a LC column and mobile phase, before ionization, mass analysis and detection by the mass spectrometer. LC-MS/MS has the added capability of simultaneous analysis of multiple compounds in a single sample and over wide dynamic ranges, which enables profiling of many metabolites of the vitamin D pathway; and importantly is the only method for vitamin D quantification with the capability to do so.

LC-MS/MS facilitates the measurement of “total” vitamin D metabolites, meaning both the metabolites bound to the VDBP and albumin, and those circulating freely. By measuring both the free and bound vitamin D metabolites, a more accurate assessment of vitamin D status can be acquired. Sample preparation methods used for LC-MS/MS analysis enable the effective release of vitamin D metabolites from the VDBP by the use of strong organic solvents and so LC-MS/MS methods measure total vitamin D metabolite concentrations.

Reliable and efficient recovery of the analyte during sample preparation is crucial as many vitamin D metabolites circulate in the low nmol/L or pmol/L range. Several sample preparation methods have been used for the extraction of vitamin D metabolites from serum and plasma, and are now being developed for other biological matrices (reviewed in [33]). With the availability of automated sample processing systems, such as the Biotage Extrahera and the Tecan Liquid Handler, sample preparation methods such as supported liquid extraction (SLE) and solid phase extraction (SPE) can be automated and are becoming more robust and reproducible, with reduced intra- and inter-batch variation in analyte recovery [261]. For the analysis of 1,25(OH)_2_D3, the use of immunoextraction prior to LC-MS/MS is becoming routine for human clinical samples [262,263,264]. Immunoextraction uses anti-1,25(OH)_2_D3 antibodies to enrich and extract this metabolite, which enhances analyte recovery from the matrix. This is beneficial due to the low circulating concentrations of 1,25(OH)_2_D3. The Vitamin D Combi ImmuTube LC-MS/MS assay (Immundiagnostik AG) kit uses this immunoextraction procedure and is discussed in Section 2 below. To the authors knowledge, there have been no reports of immunoextraction used with LC-MS/MS for the analysis of 1,25(OH)_2_D3 in veterinary species.

LC-MS/MS uses stable isotope labelled internal standards (IS) for quantitative analysis. Carbon-13 and deuterium labelled internal standards are available for most of the major vitamin D metabolites. Their equal addition to both samples, calibrators, and quality control samples (QCs) prior to sample preparation enables the use of the ratio of the response of the analyte and IS in samples for quantitation. This facilitates correction of the variation in recovery and instrument response, thereby improving the precision and accuracy of results.

**Figure 5:**
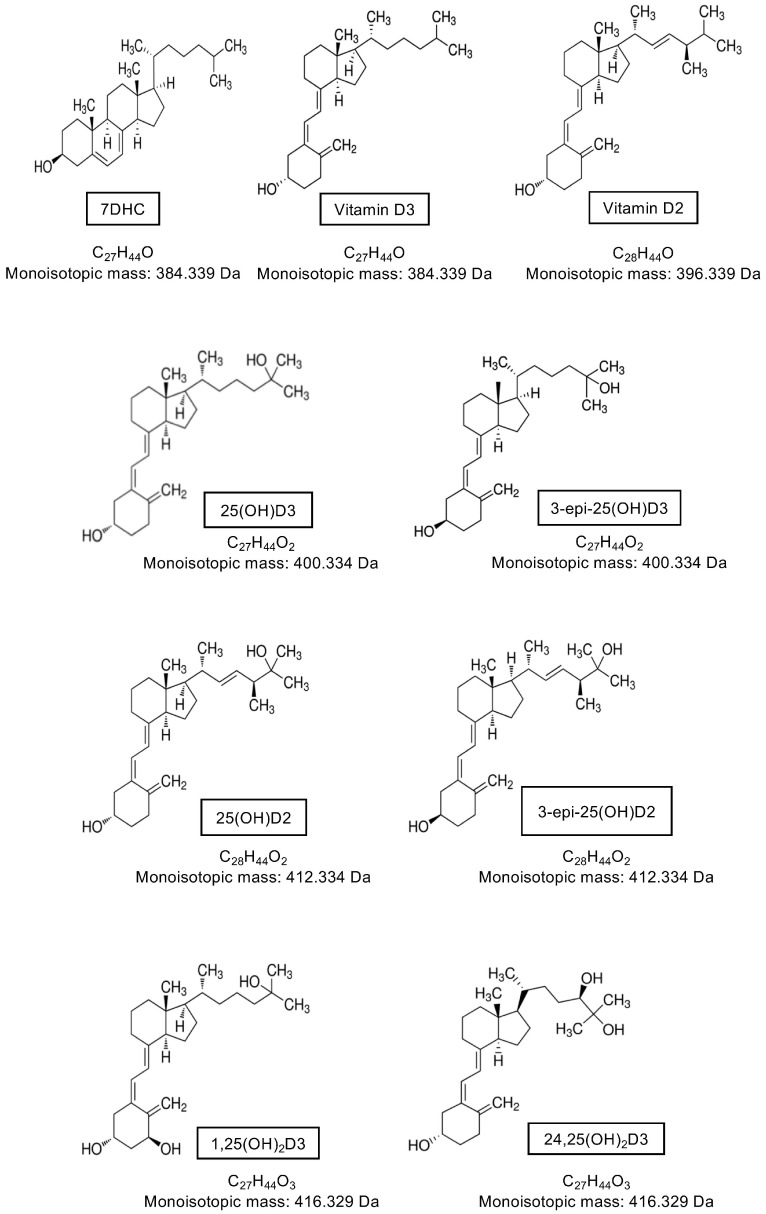
Molecular structure, formula, and monoisotopic mass of major vitamin D metabolites.

Many vitamin D molecules are very similar in chemical formulation, with similar and sometimes identical structures and weights (Figure 5). For example, only the presence of one extra carbon that forms a methyl group differentiates the D2 and D3 analytes; 1,25(OH)_2_D3 and 24,25(OH)_2_D3 have identical molecular formulas and mass but differ in structure (isomers); and the C3-epimers are identical in formula, mass and structure to the non-epimer counterpart (i.e., 25(OH)D3 and C3-epi-25(OH)D3) but differ in the orientation of the hydroxyl group at position C3, making them stereoisomers. Mass spectrometry analysis using a triple quadrupole mass spectrometer of vitamin D molecules involves ionization (usually protonation as vitamin D is ionized in positive ion mode) of the parent analyte of interest and then detection based on specific mass-to-charge (*m*/*z*) ratios. Quantitative analysis by tandem mass spectrometry uses multiple reaction monitoring (MRM), whereby the isolated parent ions are fragmented under specific collision energy settings into defined product ions—it is these product ions that are detected, identified, and used for quantification (Figure 4). As multiple vitamin D molecules are isobaric and have the same parent and product *m*/*z* ratios, they will not be differentiated by the mass spectrometer [265,266]. However, the chromatographic separation of analytes enables separation of molecules in time based on their physical and chemical interactions with the LC column and mobile phase, rather than mass and structural differences. This occurs prior to detection in the mass spectrometer and enables not only D3 and D2 metabolites to be distinguished and quantified individually, but isomers and stereoisomers such as 1,25(OH)_2_D3 and 24,25(OH)_2_D3, and the C3-epimers can be confidently distinguished given sufficient resolving power from the LC column. This resolution is critical for accurate identification and quantification, and is now possible using specialist LC columns (column chemistry, length, diameter and particle size all impact resolution capacity) and optimizing other LC parameters such as mobile phase (as well as any additives or buffers), gradient elution, flow rate, and temperature.

After chromatographic separation, the sample must be ionized and then passed into a mass spectrometer which is a highly sensitive and selective analytical detector. Electrospray Ionization (ESI) is the main ionization technique used in vitamin D LC-MS/MS methods, however some methods do report using Atmospheric Pressure Chemical Ionization (APCI) and there is at least one report of the use of Atmospheric Pressure Photoionization (APPI) [267] (see extensive review [253] regarding method details). Vitamin D metabolites do not ionize efficiently in the mass spectrometer source; they are lipophilic in nature and lack chemical functionalities with sufficient liquid phase basicity to retain a proton [253]. This lack of ionization efficiency, combined with their low abundance, means that analytical method development for vitamin D metabolites is challenging.

Many reported LC-MS/MS methods for vitamin D quantification utilize derivatization to enhance ionization efficiency and therefore, sensitivity, particularly for 1,25(OH)_2_D and 24,25(OH)_2_D metabolites [253]. Furthermore, derivatization also reduces isobaric interferences in the mass spectra by shifting the *m*/*z* range of the derivatized parent ions to a higher *m*/*z*; and depending on the derivatization reagent label, this can simplify the product ion spectra by producing products that predominately retain the label [253]. Some derivatization reagents produce 6R- and 6S- isomers, as the reagent reacts at the s-cis-diene of the compound from both the α- and β- orientations, respectively. This can result in two chromatographic peaks for every metabolite, which in multi-metabolite assays, can result in undesirably complex chromatography. It can be difficult to chromatographically resolve all the derivatized isomers whilst maintaining reasonable analysis times, meaning that there may be a compromise between sensitivity and chromatographic resolution.

Vitamin D LC-MS/MS methods are not without their analytical challenges and limitations. LC-MS instrumentation is complex, expensive to purchase and maintain, and requires technically skilled staff to operate. Workflows are not automated to the extent that immunoassays are, and method development and validation can be extensive and time consuming. Vitamin D metabolites are analytically challenging due to the presence of many highly similar compounds, low concentrations and inefficient ionization. However, it is a result of this fine tuning of the method details that provides LC-MS/MS the advantage over other methods in terms of selectivity and sensitivity.

### 4.2. Standardization of Vitamin D Analysis

One of the main challenges regarding vitamin D analysis is the lack of standardization between laboratories [268]. Harmonization of 25(OH)D testing has been challenging, with comparison studies by External Quality Assurance (EQA) programs having demonstrated substantial variability between laboratories—even between LC-MS/MS methods [269,270]. It is evident even from the non-exhaustive list of various vitamin D LC-MS/MS methods reviewed by Volmer et al. (2015) [253] just how variable vitamin D LC-MS/MS assays can be. This variability has undoubtedly impacted the ability to clarify and define reference ranges and may contribute to the conflicting results often found in research studies; as without proper standardization and calibration, comparable results between laboratories and techniques is hardly achievable.

In particular for LC-MS/MS, there is no one-size-fits-all method; laboratories use a range of different LC-MS instrumentation with differing specificities, and set up their own in-house method including sample preparation. They may use the limited available commercial assay kits (discussed below) but they are not guaranteed or dependent on certain instruments and rely upon the expertise of the analyst and laboratory to produce reliable data. However, things have improved considerably in recent years with the development of standard reference materials (SRM) [271] and reference method procedures (RMPs) [272,273,274] by the Vitamin D Standardization Program (VDSP) and EQAs such as the Vitamin D External Quality Assessment Scheme (DEQAS). The National Institute of Health (NIH) Office of Dietary Supplements established the VDSP in 2010, with the aim of standardizing the measurement of vitamin D by providing SRMs with certified reference values assigned using the NIST RMP. These are used by clinical laboratories for method validation. The main SRM currently in use for vitamin D metabolites is SRM 972a, which includes four human serum samples containing different concentrations of the following metabolites: 25(OH)D2, 25(OH)D3, 3-epi-25(OH)D3, and 24,25(OH)_2_D3. More recently, SRM 2973 has been developed, which contains higher concentrations of 25(OH)D3 and additional concentrations of 24,25(OH)_2_D3 [271]. There are currently no SRMs for the validation of other vitamin D metabolites, most notably 1,25(OH)_2_D3.

RMPs may not necessarily be suited to high-throughput clinical diagnostic or research laboratories for routine 25(OH)D analysis due to expense and time constraints. However, RMPs are invaluable to EQAs with regards to assigning reference values to serum samples for the assessment of assay performance. For example, DEQAS assigns vitamin D metabolite concentrations to human serum samples using NIST RMPs that are then dispatched to participating laboratories quarterly (five samples per quarter; four for method assessment and the fifth for DEQAS research use) and analyzed by their chosen methods [275]. Returned results of calculated metabolite concentrations are compared to the NIST RMP assigned value and also the all-laboratory trimmed mean for that method and others. This enables the participating laboratories to validate and continuously monitor their assay performance in comparison to NIST RMPs, other laboratories using the same method technique, and other methods such as immunoassays. These programs have considerably reduced the variability between laboratories [247,268] but there are still improvements to be made. SRMs will be required for other vitamin D metabolites as we discover the benefits of profiling more of the vitamin D pathway.

As the demand for vitamin D analysis by LC-MS/MS is increasing, there are now a small number of commercial assay kits available. These kits include the ClinMass LC-MS/MS Complete Kit for 25-(OH)-Vitamin D2/D3 (Recipe) and the MassChrom 25-(OH)-VitaminD3/D2 (Chromsystems). These kits typically contain sample preparation materials (plates, precipitation reagents, SPE-buffers), internal standards, QC samples, serum calibrators, and mobile phases. The MassChrom 25-(OH)-VitaminD3/D2 kit also contains a trap and an analytical LC column, but these are additional accessories for the ClinMass LC-MS/MS Complete Kit for 25-(OH)-Vitamin D2/D3. Both kits only quantify 25(OH)D metabolites (no C3-epimers, 24,25(OH)_2_D and 1,25(OH)_2_D) and they lack an internal standard for 25(OH)D2. A multiplex method kit, Vitamin D Combi ImmuTube LC-MS/MS assay (Immundiagnostik AG), is available and utilizes immunoextraction as the sample preparation method prior to LC-MS/MS. This kit can be used for the quantification of multiple metabolites including 24,25(OH)_2_D3 and 1,25(OH)_2_D3 alongside 25(OH)D2/3, and if validated would be a substantial step forward for laboratories aiming to profile vitamin D metabolites in clinical samples. The kit includes the ImmuTube -columns (which contain beads coated in the anti-vitamin D metabolite antibodies), elution and wash reagents, calibrators and control samples, internal standards, derivatization solution and mobile phases, with tuning solutions and a UPLC column available to purchase separately. There are drawbacks to using commercial assays of course. Often, they have to be used in one batch, which may not be suitable for laboratories that process low sample numbers. Even with a commercial kit, the assay still requires validation in each individual laboratory which can be time consuming to set up and troubleshoot. Although some kits can provide everything required for the assay from mobile phase to the LC column, many laboratories may prefer to use some their own consumables for laboratory consistency and for economic reasons. This could introduce unknown factors into the assay and may impact kit performance. Given the range of LC-MS instrumentation available, it is also likely that a laboratory may have a different LC-MS instrumentation set up compared to the kit manufacturer, which again could impact kit performance and validation requirements. The nature of LC-MS/MS methods means that even with commercial kits, standardization is not fully achieved.

There are currently no kits or EQAs for the comprehensive analysis of vitamin D specific for veterinary species. With the current veterinary research literature demonstrating a similar association between vitamin D status in health and disease as in humans, there is increasing demand for the assessment of vitamin D metabolites in veterinary species, both for research purposes and in the clinics. There is a real need for a comprehensive, reliable method that is comparable to that used in human clinical practice for the assessment of vitamin D metabolites in veterinary species, and critically to enable the establishment of reference ranges. Limited studies have begun to develop and apply LC-MS/MS methods specifically to the species of interest in the study (Table 1) [80,81,84].

### 4.3. Challenges in Veterinary Vitamin D Analysis

#### 4.3.1. Sample Acquisition and Preparation

There are particular challenges for the bioanalysis of vitamin D metabolites in clinical samples from veterinary species. In human clinical laboratories and research trials with more standardized procedures, there are set requirements for sample type, volume, and other preanalytical requirements including storage temperatures; however, this can be more variable in the veterinary field, especially outside of clinical practice (samples from zoo animals, for example). This is due to samples being more challenging to acquire, and they are subject to more extensive ethical consideration. Sample volume can vary greatly between species, with very small volumes from rodents to larger volumes available from large animals such as cows and horses. This can impact sample preparation methods which are generally optimized for a set volume. If the required volume is not available and samples have to be diluted, dilution integrity should be demonstrated by accuracy and precision parameters of QCs during validation [276,277]. Most vitamin D LC-MS/MS methods require serum samples for analysis, but due to constraints on blood sampling plasma may be the only available matrix in some veterinary cases. Anticoagulants commonly used in blood collection tubes for plasma samples include ethylenediaminetetraacetic acid (EDTA), heparin and citrate, and these preanalytical factors can impact analyte recovery [278]. This impact is analyte dependent, and although few studies investigating 25(OH)D concentrations in human plasma suggest that the effect of anticoagulants on this metabolite is minimal [279,280], the impact of various anticoagulants on analyte recovery should be validated for veterinary species and for other vitamin D metabolites.

The matrix used for calibration standards for the assay should also be carefully considered. Recommended guidelines state this should be the same matrix as the sample being analyzed, but stripped of the analyte of interest. However, vitamin D stripped serum is not available for many species. Surrogate matrices such as bovine serum albumin (BSA) may be used, and if so it is important to consider the concentration used. Human serum contains higher concentrations of albumin in comparison to cats and dogs, with a reference range between 35 to 55 g/L for humans; whereas the reference range for canine serum albumin is approximately 27 to 38.8 g/L and feline serum albumin is 28 to 39 g/L [281]. The concentration of BSA used in calibrators should therefore be optimized for each species in terms of analyte recovery and matrix effects, with the aim that the calibration solution should closely represent the real sample matrix. Commercially available surrogate matrices are becoming available for human samples, for example SigMatrix Serum Diluent (Sigma-Aldrich). Currently, there are no commercially available alternatives for veterinary species. Furthermore, differences in the matrix components between species must be considered [282,283,284]. Endogenous compounds such as proteins and phospholipids, and exogenous compounds such as veterinary drugs and medications can produce matrix effects which differ between species.

#### 4.3.2. Assay Modification and Validation for Use in Veterinary Species

Differences in vitamin D metabolite profiles can impact the applicability of an assay. Animals exhibit different baseline concentrations of vitamin D metabolites in comparison to humans and each other, for example: dogs have high 25(OH)D3 concentrations, horses generally have very low vitamin D status, and cats have high levels of C3-epimers which need to be chromatographically separated. If the concentration range of vitamin D metabolites in a particular species sample is unknown (which is likely due to the lack of reference ranges for many species) broad calibration ranges will be required for initial testing in order to determine what concentration the various metabolites are measuring at. For subsequent analysis, the calibration range may be narrowed to improve accuracy and precision.

Guidelines for the validation of bioanalytical assays for endogenous compounds are somewhat limited [285]. Those developing LC-MS/MS or ligand-binding assays for endogenous biomarkers are generally guided by recommendations for the analysis of exogenous compounds, such as those from the Food and Drug Administration (FDA) and the European Medicines Agency (EMA) [276,277]. In drug discovery, bioanalytical methods by design must switch between species matrices; most studies begin in rodents, then transfer to dogs and then onto humans for clinical trials. The guidelines for transferring a bioanalytical method for exogenous compounds from one species to another recommend that partial validations are conducted to validate the modifications of the previously fully validated method [286,287]. Partial validations are performed to assess the validity of the modification of the method; the modification in this case would be a change of species in the matrix. Parameters to be evaluated in the partial validation should be selected considering the potential impact of the modifications on the method, and can range from one intra-assay accuracy and precision determination to a nearly full validation [277,286]. Unfortunately, no specific guidance is provided for each type of modification. However, given the potential variability of the matrix between species, and therefore differences in matrix effects, this type of modification is considered significant [287].

The potential significance of the impact of differing matrix effects suggests that it would be reasonable to include such parameters (matrix effects, recovery, etc.) during partial validation for a change in species matrix. Matrix effects occur when ionization of the desired ion is perturbed by undetectable components in the sample matrix that coelute with the analyte(s) of interest [286]. This results in reduction (ion suppression) or enhancement (ion enhancement) of the ion intensity of the analyte of interest, and ultimately significantly reduces the accuracy and precision of the assay [286,288]. The impact of matrix effects on LC-MS/MS methods and how to reduce them have been well reviewed [289,290,291,292,293]. To determine the selectivity of the current assay for the new species matrix (i.e., the ability of the analytical method to differentiate and quantify the analytes of interest in the presence of interfering matrix components), it is recommended to analyze blank sources of the appropriate matrix for interferences and ensure selectivity is assured at the lower limit of quantitation (LLOQ), using matrix from at least six sources [286]. The FDA guidelines define interference as a peak response in blanks or zero standards at or equal to 20% of the LLOQ response [277,286]. One of the challenges for veterinary species is that there is often a limited amount of matrix available for assay testing.

The matrix factor (MF) can be utilized to ascertain matrix effects. MF is defined as the ratio of the analyte response in the presence of matrix ions to analyte response in the absence of matrix ions [286,294]. The MF in the absence of matrix ions should be 1, and in the presence of matrix a value above or below 1 indicates ion suppression or enhancement, respectively. It is the variability of the MF within a species that is often of most concern, and it is suggested that variability in MF should be determined in six different matrix lots, with an acceptable variability of <15% [294]. The EMA guidance recommends that the variability of the IS-normalized MF (analyte to IS ratio in the matrix extracts divided by the analyte to IS ratio in the absence of matrix extract) from six lots of matrix should not be greater than 15% at low and high QC concentrations [276].

Recovery of the analyte during sample preparation can also be affected by matrix components and as mentioned above this is an important aspect to consider for vitamin D metabolite analysis. Recovery should be assessed in the new species matrix by comparing analyte extracted from samples at three concentrations (low, medium, and high) with unextracted standards at the same concentrations representing 100% recovery. Although recovery does not need to be 100%, the recovery of the analyte and internal standard must be consistent and reproducible [286]. If low recovery is impacting on assay sensitivity for a particular species matrix, sample preparation should be optimized.

Using stable isotopically-labelled internal standards (Sil-IS) can compensate for differences between calibrator and sample matrices [295] and moderate matrix effects. SIL-ISs are available for the major vitamin D metabolites (25(OH)D2/3, C3-epi-25(OH)D2/3, 24,25(OH)_2_D3, and 1,25(OH)_2_D3). Equal amounts of the SIL-IS is added to both samples and calibrators before sample preparation, thereby undergoing the same extraction and analytical pressures. A good SIL-IS is required to show almost identical behaviour to the analyte of interest during chromatography and ionization, with the same retention time, therefore undergoing the same matrix effects. Using the response ratio of the analyte: SIL-IS in both the calibrators and samples will compensate for differences between matrices, matrix effects and variations in recovery during sample preparation.

The use of ion ratios as part of the result assessment can aid in the identification of potential interferences from the matrix. For each parent ion (i.e., each vitamin D metabolite of interest) at least two product ions are assigned; one quantitative product ion which will be used for quantification purposes, and at least one qualitative product ion which is used to confirm the identity of the analyte (Figure 4). The peak response of the quantitative and qualitative product ions are calculated as a ratio that is monitored for each transition. This ratio should be consistent for every sample and standard, with an acceptable error of 15% to 20%. If the ratio of the quantitative:qualitative peak response differs above this threshold it suggests potential interference from the matrix. Consistent monitoring of quantitative:qualitative ratios will therefore help to identify any matrix interferences when a new type or species of matrix is assessed.

For the optimal assessment of vitamin D in veterinary species, a vitamin D LC-MS/MS method would be initially validated for one species and then further partially validated for all other species it is to be utilized for. If fortunate, the assay may be suitable to use in multiple species without adaptation; however, if the partial validation reveals that some method parameters do not meet requirements it may be necessary to amend parts of the method and essentially have different methods for different species. Realistically, it is unlikely to be feasible to obtain enough samples of each veterinary species requiring analysis that would allow for extensive validation. If it is not possible to validate methods for all species, a one-size-fits-all approach may be the next best option. If the assay is validated for one or even two species that samples are available for, would it be sufficient to apply this method to other species and monitor performance via the use of a good SIL-IS and ion ratios? Would a simple sample preparation method be all that is necessary if a good SIL-IS and ion ratios are utilized or should sample extraction be extensive to remove as many interferences from the matrix as possible? Studies investigating the performance of different validation methods across species are needed to fully understand how LC-MS/MS methods can be feasibly adapted to multiple species.

### 4.4. Analysis of Vitamin D from Non-Invasive Biological Matrices

The development of non-invasive tests for animal species is highly beneficial, particularly for wild or endangered species, but also in a clinical setting to reduce stress and risk to the animal. The development of non-invasive endocrine tests has been of particular interest in terms of assessing stress in animals, and is of relevance to vitamin D as the assessment of steroid hormones such as glucocorticoids is analytically very similar [296,297,298]. Although vitamin D metabolites are routinely assessed from serum and plasma, there have been a few publications investigating the feasibility of assessing vitamin D metabolites in non-invasive biological matrices, for example urine [299,300,301], saliva [302,303,304], and hair [305], but currently no studies have been conducted in veterinary species.

In humans, a method described by Carlow et al. (2016) for the assessment of 25(OH)D2 and 25(OH)D3 in urine was unable to detect the metabolites in healthy adult urine with a LOQ of 50 pmol/L (20 pg/mL), but detected higher concentrations of the metabolites in urine from patients with nephrotic syndrome [299]. Higashi et al. (2002) and Ogawa et al. (2014) report a more sensitive method and were able to detect 23,25(OH)_2_D3 and 24,25(OH)_2_D3, and 25(OH)D3 and 24,25(OH)_2_D3, respectively, with lower LOQs when utilizing derivatization and pre-treating the urine with β-glucuronidase [300,301]. Glucuronidation occurs in vitamin D metabolites across the CYP24A1 pathway, and glucuronide conjugated metabolites have been demonstrated to be excreted in the bile of dogs and rats [9]. Glucuronide metabolites are highly polar and cannot be easily retained in reverse phase chromatography separation, hindering the quantitative ability of the analytical method. Pre-treatment of the urine samples with β-glucuronidase hydrolyses the glucuronide conjugated metabolites back to the native parent compound, allowing for the accurate assessment by LC-MS/MS. By doing so, Ogawa et al. (2014) were able to detect and quantify 25(OH)D3 and 24,25(OH)_2_D3 in human adult urine relative to creatinine concentration [301]. Further investigation is needed to determine how the vitamin D metabolite concentrations in urine correlate to those in circulation as both Ogawa et al. and Higashi et al. noted differences in the ratios of measured metabolites in urine in comparison to what is normal in serum. Higashi et al. reported approximately equal concentrations of 23,25(OH)_2_D3 and 24,25(OH)_2_D3 in urine, whereas in human serum, C24 hydroxylation is much more common and 24,25(OH)_2_D is detected at higher levels than 23,25(OH)_2_D3 [300]. Ogawa et al. reported much higher concentrations of 24,25(OH)_2_D3 in urine than 25(OH)D3, highlighting the catabolic pathway of vitamin D, whereas in serum 25(OH)D3 circulates at much higher concentrations than 24,25(OH)_2_D3 in humans [301]. The metabolite profiles would need to be elucidated in both serum and urine for many species to allow comparisons to be made. Urine would be a much easier sample to obtain from animal species, and at greater volumes. On the contrary, a more sensitive LC-MS/MS method will likely be required due to the anticipated much lower concentrations of metabolites in urine.

Methods have been described for the LC-MS/MS analysis of 25(OH)D from saliva which would be useful for patients in which it is difficult to obtain a blood sample, for example young children and animals. Although vitamin D metabolites were detectable in saliva samples by LC-MS/MS, there are many factors which appear to influence the concentration of 25(OH)D in saliva, including saliva flow rate, how the sample is collected (passively via drooling versus stimulated by a swab or chewing gum), the VDBP presence and contaminants such as mucin. Clarke et al. (2019) reported that the best results were achieved when daily measurements were obtained across three consecutive days. Although it may provide painless assessment of vitamin D status, multiple steps are required for sample preparation and until the confounding factors can easily be corrected for, this method in its current state would not be suitable for cohort studies and could be challenging to apply in the field [302].

A proof-of-concept study by Zgaga et al. (2019) is the first to demonstrate the ability to quantify 25(OH)D3 in human hair samples. Results were compared to those from serum samples obtained at the same time, and show that 25(OH)D3 concentration was much more variable from hair samples than from serum [305]. The relationship between serum and hair concentrations will have to be determined, as will factors which influence the deposition of 25(OH)D3 in hair and factors which may impact its extraction. The author stipulates that as hair provides a period measurement with lag time in comparison to blood sampling, which provides a point-measurement of vitamin D status, there may not be a significant correlation and recommends hair measurement not to be a replacement for blood measurement, but a useful adjunct to provide long-term information on vitamin D status [305]. This temporal assessment of vitamin D could be useful in longitudinal studies and in epidemiological research, and may be particularly useful for long-term vitamin D assessment of farmed animals.

## 5. Conclusions

The demand for vitamin D analysis in veterinary species is increasing as our knowledge of its extra-skeletal roles in health and disease continues to grow. However, the veterinary field is clearly behind in its approach to vitamin D metabolite assessment, with few studies using the gold-standard method of LC-MS/MS for measurement. In this review, we have highlighted the current known differences in vitamin D metabolism between veterinary species, and discussed cases in which profiling vitamin D metabolites beyond the standard assessment of 25(OH)D has already benefitted veterinary patients. The adoption of the only multi-metabolite analytical approach, LC-MS/MS, for the analysis of vitamin D will provide a more informative profile of this complex and dynamic pathway; this will not only provide further insight into species differences in vitamin D metabolism, but will highlight differences in metabolic profiles between patient/disease groups, which ultimately may be used as complex biomarkers in prognostic and diagnostic applications. Better study designs and more robust analytical methods will be required with specific validation for veterinary species, especially as we become more aware of species differences in vitamin D metabolic profiles. The establishment of reference ranges in veterinary species using a single multi-metabolite method, along with increased transparency when documenting methods, is critical in order to make studies more comparable between laboratories. This will be required in order to bridge the gap in knowledge between vitamin D intake and status in veterinary patients. Ultimately, the aim is to further our understanding of this complex pathway in both health and disease, in order to manipulate it more efficiently using a treat-to-target approach. Profiling the vitamin D pathway will enable a more efficient investigation into the effectiveness of vitamin D supplementation, and will hopefully begin to clarify some of the conflicting results reported in both human and veterinary literature.

## Figures and Tables

**Table 1:** Details of reported LC-MS/MS assays for the analysis of veterinary samples.

Citation	Species	Metabolites Measured	LOQ	*m*/*z* Transitions	Sample Type/Vol	Sample Prep Method	Derivatization	LC Column	Ionization Source	LC-MS System
Hurst (2020) [80]	Dog	25(OH)D2 25(OH)D3	0.5 nmol/L 4.0 nmol/L	758.4 -> 740.2 747.3 -> 729.3	Serum/200 μL	SLE	Yes—DMEQ-TAD	Raptor FP (2.7 μm, 100 × 2.1 mm)	ESI	Shimadzu Nexera X2 UHPLC—Sciex QTrap 6500+
Hurst (2020) [81]	Dog	25(OH)D2 C3-epi-25(OH)D2 25(OH)D3 C3-epi-25(OH)D3	7.8 nmol/L 7.8 nmol/L 4.0 nmol/L 4.0 nmol/L	413.3 -> 395.3 413.3 -> 395.3 401.3 -> 383.3 401.3 -> 383.3	Serum/200 μL	SLE	No	Raptor FP (2.7 μm, 100 × 2.1 mm)	ESI	Shimadzu Nexera X2 UHPLC—Sciex QTrap 6500+
Groth (2019) ^b^ [82]	Dog	25(OH)D3 24,25(OH)_2_D3	Not specified	Not specified	Serum/vol not specified	Not specified	Not specified	Not specified	Not specified	Agilent 1290 HPLC—Agilent 6460 triple quadrupole
Mick (2019) ^b^ [83]	Dog	25(OH)D3 24,25(OH)_2_D3 1,25(OH)_2_D3	Not specified	Not specified	Serum/vol not specified	Not specified	Not specified	Not specified	Not specified	Agilent 1290 HPLC—Agilent 6460 triple quadrupole
Fritz (2017) [84]	Rat Dog Mouse Monkey	25(OH)D3 25(OH)D2	5.0 nmol/L	383.3 -> 229.2 395.3 -> 269.3	Serum/50 μL	SLE	No	Phenomenex Luna C8 (3 μm, 5 0 × 2.0 mm)	APCI	Agilent HPLC—Sciex 4000 QTrap
Sprinkle (2017) ^d^ [85]	Cat Dog Rat	25(OH)D3 C3-epi-25(OH)D3	Not specified	401.4 -> 383.3 401.4 -> 383.3	Serum/200 μL	PPT + online extraction	No	Chirex-PGLY and DNB (250 × 4.6 mm)	APCI	TX4 Turbo Flow—Sciex API 4000
Weidner (2017) ^b^ [28]	Dog	24,25(OH)_2_D3	Not specified	Not specified	Serum/vol not specified	Not specified	Not specified	Not specified	Not specified	Agilent 1290 HPLC—Agilent 6460 triple quadrupole
Willcox (2016) ^c^ [86]	Dog	25(OH)D2 25(OH)D3	12.5 nmol/L	413 -> 395 401 -> 383	Serum/100 μL	SPE	No	Not specified	Not specified	Not specified
Spoo (2015) ^a^ [87]	Dog	25(OH)D3 24,25(OH)_2_D3	Not specified	Not specified	Serum/plasma 100 μL for 25(OH)D3 200 μL for 24,25(OH)2D3	PPT + LLE	No	Not specified	Not specified	Agilent 1290 HPLC—Agilent 6460 triple quadrupole
Azarpeykan (2016) ^e^ [88,126]	Horse	25(OH)D2 25(OH)D3	<4 nmol/L	413.3 -> 355.4 401.3 -> 383.4	Serum/60 μL	PPT + LLE	No	ACE C8 (3 μm, 50 × 2.1 mm)	ESI	Shimadzu HPLC—Sciex 4000
Allott (2020) ^e^ [89]	Sheep	25(OH)D3 25(OH)D2	Not specified	Not specified	Plasma/vol not specified	Not specified	Not specified	Not specified	Not specified	Not specified
Dittmer (2020) ^e^ [90]	Sheep	25(OH)D3 25(OH)D2	Not specified	Not specified	Serum/vol not specified	Not specified	Not specified	Not specified	Not specified	Not specified
Zhou (2019) [91]	Sheep	25(OH)D2 25(OH)D3	7.2 nmol/L 5.6 nmol/L	758.5 -> 740.0 746.5 -> 728.4	Serum/100 μL	PPT + SPE	Yes—DMEQ-TAD	ACE UltraCore 2.5 SuperC18 (2.5 μm, 75 × 2.1 mm)	ESI	Ultimate 3000 HPLC—Bruker amaZon ETD
Handel (2016) ^f^ [30]	Sheep	25(OH)D2 25(OH)D3	Not specified	Not specified	Serum/vol not specified	Not specified	Not specified	Not specified	Not specified	LC system not specified—Sciex QTrap 5500
Dittmer (2011) ^e^ [202]	Sheep	25(OH)D3	Not specified	Not specified	Serum/vol not specified	Not specified	Not specified	Not specified	Not specified	Not specified
Celi (2018) ^h^ [92]	Cattle	Vitamin D2 Vitamin D3 25(OH)D3	Not specified	Not specified	Serum tissues (fat, muscle, kidney, liver) Feedstuff	Not specified	Not specified	Not specified	Not specified	Not specified
Guo (2018) ^g^ [93]	Cattle	Vitamin D3 25(OH)D3	Not specified	Not specified	Plasma/100 μL Milk/3 g for vitamin D3 and 8 g for 25(OH)D3	PPT + LLE for plasma Saponification + LLE for milk vitamin D3 Saponification + LLE + SPE for milk 25(OH)D3	No	Not specified	APCI	Agilent 1290 HPLC—Sciex 4000
Alexander (2017) ^b^ [94]	Pig	Vitamin D3	Not specified	Not specified	Serum/vol not specified	Not specified	Not specified	Not specified	Not specified	Agilent 1290 HPLC—Agilent 6460 triple quadrupole
Flohr (2016) ^i^ [157,158]	Pig	Vitamin D3 25(OH)D3 Vitamin D3 (feed)	2.5 nmol/L 12.5 nmol/L 1.6 μg/kg	Not specified 383 -> 159 Not specified	Serum/vol not specified Foodstuff/amount not specified	LLE for serum Saponification + LLE for foodstuff	No	Serum—Zorbax Eclipse XDB-C18 (5 μm, 150 × 4.6mm) Foodstuff—2D LC with Ascentis Express octylsilyl (C8) (2.7 μm, 150 × 3 mm) and Ascentis Express C18 (2.7 μm, 150 × 3mm)	Serum—ESI Foodstuff—ESI (Turbo Ion Spray)	Serum—Agilent 1200 HPLC—Agilent 6410 triple quadrupole Foodstuff—Agilent 1200 HPLC—Sciex API 4000
Kuhn (2015 + 2014 + 2019) [95,175,176]	Poultry	25(OH)D3 Vitamin D3 25(OH)D3	3.7 nmol/L 0.17 μg/100g 0.1 μg/100g	576 -> 298 560 -> 298	Plasma/vol not specified Egg yolk + skin	LLE Saponification + LLE + HPLC	Yes—PTAD	Hypersil ODS (5 μm, 100 × 2 mm)	ESI	Agilent 1100—API 2000 (Applied Biosystems)
Browning (2014) ^j^ [172]	Poultry	Vitamin D3 25(OH)D3	0.1 μg/kg	Not specified	Egg yolk/7.5 g	Saponification + SPE	Not specified	Not specified	Not specified	Not specified
Schutkowski (2013) [96]	Poultry	25(OH)D3 Vitamin D3 25(OH)D3 7-DHC	3.7 nmol/L 0.17 μg/100g 0.1 μg/100g Not specified	Not specified	Plasma/vol not specified Egg yolk + meat/vol or weight not specified	LLE Homogenization and LLE	Yes—PTAD	Hypersil ODS (5 μm, 100 × 2 mm)	Not specified	Agilent 1100—API 2000 (Applied Biosystems)
Ziegler (2018) [98]	Baboon	Vitamin D3 25(OH)D2 25(OH)D3 24,25(OH)2D3	Not specified 2.19 nmol/L 2.52 nmol/L 1.15 nmol/L	Not specified 413.3 -> 355.3 401.3 -> 159.0 417.3 -> 121.0	Serum/100 μL	PPT + SPE	Yes—PTAD	Phenomenex Kinetex C18 (2.6 μm, 100 × 2.1 mm)	ESI	Shimadzu Prominence HPLC—Sciex QTrap 5500
Ziegler (2015) [97]	Marmosets Rhesus macaques	25(OH)D2 25(OH)D3 1,25(OH)2D3	1.3 nmol/L 37.5 pmol/L	Not specified 748.6 -> 689.5	Serum/50 μL for rhesus macaques and 10 μL for marmosets Serum/200 μL	PPT Dual column SPE	No Yes—Amplifex	Phenomenex Luna C8 (3 μm, 50 × 2 mm) Phenomenex Kinetex C18 (2.4 μm, 150 × 3 mm)	APCI ESI (Turbo Spray Ion)	Shimadzu Prominence HPLC—Sciex QTrap 5500 Shimadzu Prominence HPLC—Sciex QTrap 5500
Kale (2018) ^e^ [254]	Brown kiwi, Tuatara, New Zealand Sea Lion	25(OH)D2 25(OH)D3	Not specified	Not specified	Plasma + serum/vol not specified	Not specified	Not specified	Not specified	Not specified	Not specified

LC-MS/MS (liquid chromatography tandem mass spectrometry); LOQ (limit of quantification); *m*/*z* (mass-to-charge) transition; PPT (protein precipitation); LLE (liquid–liquid extraction); SLE (supported liquid extraction); SPE (solid phase extraction); ESI (electrospray ionization): APCI (atmospheric pressure chemical ionization); LC column dimensions (particle size, length × I.D.). Where details are not specified a superscript letter denotes that samples were sent to external laboratory for analysis and details were not provided: ^a^ states that method outlined by Agilent, ^b^ Heartland Assays (Ames, IA, USA), ^c^ McClendon Clinical Laboratories, UNC Hospitals (details from referenced [255]), ^d^ Mayo Laboratories (details from referenced [256]), ^e^ Endolab (Canterbury Health Laboratories, Christchurch, New Zealand) (details from referenced [257] and [258]), ^f^ Supraregional Assay Service Laboratory, ^g^ plasma analysis was conducted by DSM Nutritional Products Ltd. (Kaiseraugst, Switzerland) and milk analysis was conducted by RTC (Pomezia, Italy), ^h^ states only that analysis was done by LC-MS/MS but provides no further information, ^i^ analysis was conducted by DSM Nutritional Products Ltd. (Kaiseraugst, Switzerland) following methods outlined by [259] for foodstuff analysis and [260] for serum analysis, and ^j^ Australian Government National Measurement Institute. Where the LOQ was provided in mass units, it was converted to molar mass by the following conversion factors: 2.496 for monohydroxy metabolites (25(OH)D) and 2.4 for dihydroxy metabolites (1,25(OH)_2_D), and listed to one decimal place.
